# Nanoencapsulation of Essential Oils for Food Preservation and Agricultural Applications: A Systematic Review of Biological Efficacy, Safety, and Technological Challenges

**DOI:** 10.3390/ijms27209154

**Published:** 2026-10-15

**Authors:** Sara Diogo Gonçalves, Luís R. Silva

**Affiliations:** 1 Gabinete de Projetos e Investigação Aplicada, 5090-150 Murça, Portugal; 2 Escola Superior de Saúde, Universidade Politécnica de Viseu, 3504-510 Viseu, Portugal; 3 RISE-Health, Faculty of Health Sciences, University of Beira Interior, Av. Infante D. Henrique, 6200-506 Covilhã, Portugal

**Keywords:** nanoformulation, nanoemulsions, antimicrobial activity, active packaging, nanocarriers, plant-derived bioactives, controlled release, food safety, agricultural protection, phytotoxicity

## Abstract

Essential oils (EOs) are promising natural bioactive compounds for food preservation and sustainable agriculture because of their antimicrobial, antifungal, antioxidant, and insecticidal properties. However, poor water solubility, high volatility, and chemical instability limit their practical application. Nanoencapsulation has emerged as an effective strategy to improve EOs stability, bioavailability, controlled release, and biological performance. This systematic review evaluated the efficacy, safety, and technological challenges of nanoencapsulated EOs for food and agricultural applications. The review followed PRISMA 2020 guidelines. Scopus, PubMed, and Web of Science were systematically searched, and original experimental studies reporting at least one safety-related outcome were included. Risk of bias was assessed using the Quality Assessment Tool for In Vitro Studies, and results were synthesized narratively. Of 776 records identified, 19 studies met the inclusion criteria. Nanoencapsulation generally improved EOs stability, controlled release, and biological persistence while frequently enhancing antimicrobial, antifungal, antioxidant, insecticidal, and preservation efficacy. Preliminary safety assessments, including cytotoxicity, hemolysis, phytotoxicity, sensory evaluation, and nanoparticle migration, were generally favourable. However, methodological heterogeneity, predominantly laboratory-based evidence, and limited long-term toxicity and industrial validation reduce the certainty of the evidence. Overall, nanoencapsulation represents a promising approach for enhancing the practical application of EOs, although standardized safety assessments and large-scale validation studies are needed before widespread commercial implementation.

## 1. Introduction

Essential oils (EOs) are complex mixtures of volatile secondary metabolites produced by aromatic plants and have attracted considerable interest as natural alternatives to synthetic antimicrobial agents, antioxidants, preservatives, and pesticides [1]. Their diverse biological activities mainly stem from terpenes, terpenoids, phenylpropanoids, and other bioactive compounds that can disrupt microbial membranes, inhibit oxidative processes, and interfere with multiple cellular and metabolic pathways. These properties have driven growing research into applying EOs in food preservation, active packaging, postharvest disease management, and sustainable agriculture [2,3]. Moreover, growing consumer demand for clean-label foods and increasing concerns about antimicrobial resistance, chemical pesticide residues, and the environmental impact of synthetic additives have further accelerated interest in plant-derived bioactive compounds as safer, more sustainable alternatives [4].

Despite their promising biological activities, several physicochemical limitations constrain the practical application of EOs. Most EOs are highly volatile, poorly water-soluble, chemically unstable, and susceptible to degradation upon exposure to oxygen, light, moisture, and elevated temperatures [5]. These properties can compromise stability and bioavailability, shorten duration of action, and require relatively high concentrations to achieve effective antimicrobial activity. Such concentrations may, however, adversely affect food sensory properties or increase the risk of phytotoxicity when EOs are applied in agricultural systems. Consequently, considerable research has focused on developing advanced delivery systems to enhance EO stability and bioavailability, enable controlled and sustained release, and improve overall efficacy while minimizing undesirable sensory and toxicological effects [6].

Nanotechnology has emerged as a promising strategy for overcoming these limitations. Nanoencapsulation involves incorporating EOs into nanoscale delivery systems, including nanoemulsions, polymeric nanoparticles, liposomes, nanocapsules, Pickering emulsions, mesoporous silica nanoparticles, and other nanocarriers. These systems can protect volatile and chemically labile constituents from environmental degradation, improve aqueous dispersibility and physicochemical stability, enhance bioavailability, and facilitate controlled or sustained release. By modulating the physicochemical properties and release kinetics of EOs, nanoencapsulation can improve their efficacy while reducing the concentrations required for antimicrobial or other biological effects, thereby potentially minimizing undesirable sensory, phytotoxic, and environmental impacts [7]. These delivery systems may also enhance the interaction of EO constituents with microbial cells, facilitating penetration into or disruption of microbial membranes and potentially improving antimicrobial efficacy while reducing the effective dose required. Consequently, nanoencapsulated EOs have attracted increasing interest for a broad range of applications, including edible coatings, active food packaging, natural food preservation, postharvest disease management, botanical pesticides, and more sustainable crop protection strategies [8].

Over the past decade, research on nanoencapsulated EOs has expanded rapidly, with numerous experimental studies reporting enhanced antimicrobial, antifungal, antioxidant, insecticidal, and preservation-related activities compared with conventional formulations [6,9,10,11]. However, the available evidence remains heterogeneous. Studies differ substantially in the botanical sources and chemical composition of the EOs, encapsulation materials and nanocarrier systems employed, physicochemical characterization approaches, biological models, experimental conditions, and outcome measures. This methodological and formulation variability makes direct comparisons across studies challenging and limits the generalizability of reported findings [12]. Furthermore, although many studies report improved biological performance, few directly compare nanoencapsulated formulations with the corresponding free EOs, making it difficult to determine the extent to which nanoencapsulation provides a genuine added benefit [13]. Safety assessment also varies widely across the literature, ranging from basic cytotoxicity assays to more comprehensive evaluations of hemolysis, phytotoxicity, sensory acceptability, nanoparticle migration, and environmental compatibility. Moreover, long-term toxicological data, harmonized safety assessment protocols, industrial-scale validation, and clear regulatory frameworks remain limited. These gaps remain important barriers to translating promising laboratory findings into safe, reproducible, and commercially viable applications [13].

These uncertainties have fuelled ongoing debate about the balance between the technological benefits of nanoencapsulation and the potential safety, environmental, manufacturing, and regulatory challenges associated with using nanomaterials in food and agricultural systems. Although nanoencapsulation is increasingly recognized as an effective strategy for improving the delivery, stability, and performance of EOs, important questions remain concerning scalability, production costs, long-term formulation stability, environmental fate and persistence, consumer acceptance, and regulatory approval [14]. Addressing these knowledge gaps requires a systematic, critical evaluation of the efficacy and safety of existing EO nanoformulations, along with greater harmonization of characterization, testing, and risk-assessment approaches to support responsible translation from laboratory research to practical applications.

Therefore, this systematic review aimed to critically evaluate the available evidence on the biological efficacy, safety, and technological challenges associated with nanoencapsulated EOs for food preservation and agricultural applications. Specifically, the review characterizes the main nanoformulation strategies reported to date, examines their antimicrobial, antifungal, antioxidant, insecticidal, and preservation-related performance, evaluates the safety assessment approaches used across studies, and identifies key limitations, knowledge gaps, and priorities for future research.

Overall, the available evidence suggests that nanoencapsulation can substantially enhance the technological applicability of EOs by improving their physicochemical stability, dispersibility, controlled release, and biological performance. However, considerable variability in formulation strategies, characterization methods, experimental models, and safety assessment approaches limits direct comparison across studies and the generalizability of the findings. Further standardized safety evaluation, robust comparative studies, industrial-scale validation, and harmonized regulatory frameworks are therefore needed to facilitate the safe, effective, and sustainable translation of EO nanoformulations into commercial food preservation and agricultural applications.

## 2. Methods

### 2.1. Research Question

The research question was developed using the Population, Intervention, Comparator, Outcomes, and Study design (PICOS) framework to ensure a structured, reproducible review process [15].

- Population (P): Food- and plant-related experimental models evaluating EOs.
- Intervention (I): Nanoencapsulated EOs, including nanoemulsions, liposomes, polymeric nanoparticles, solid lipid nanoparticles, nanostructured lipid carriers, and other nanocarrier systems.
- Comparator (C): Non-encapsulated (free) EOs, conventional formulations, untreated controls, or alternative nanoencapsulation systems, where applicable.
- Outcomes (O): Biological efficacy, including antioxidant, antimicrobial, and antigenotoxic activities; safety outcomes, including cytotoxicity, phytotoxicity, and environmental toxicity; and technological characteristics, such as particle size, encapsulation efficiency, physicochemical stability, controlled release, and bioavailability.
- Study design (S): Original experimental studies (in vitro, ex vivo, and in vivo) published in peer-reviewed journals.

Based on the PICOS framework, the primary research question was as follows:

Does nanoencapsulation improve the biological efficacy and safety of EOs compared with their non-encapsulated forms in food and plant applications, and what are the main technological challenges limiting their practical implementation?

The review was conducted in accordance with the Preferred Reporting Items for Systematic Reviews and Meta-Analyses (PRISMA) 2020 guidelines and was prospectively registered in the Open Science Framework (OSF) (2026, doi:10.17605/OSF.IO/7XVFA), an international database of prospectively registered systematic reviews, and is publicly available [16].

### 2.2. Eligibility Criteria

The eligibility criteria were established a priori according to the review objectives and the PICOS framework. Two reviewers screened studies independently based on titles, abstracts, and full texts. Eligible studies were then grouped by nanoencapsulation system, application (food or plant), experimental model, and outcome measures to facilitate evidence synthesis.

#### 2.2.1. Inclusion Criteria

Studies were considered eligible if they met all of the following criteria:

▪ Original peer-reviewed experimental research articles.
▪ Studies evaluating EOs formulated as nanoencapsulated or nanodispersed delivery systems, including, but not limited to, nanoemulsions, liposomes, polymeric nanoparticles, chitosan nanoparticles, solid lipid nanoparticles, nanostructured lipid carriers, nanocapsules, Pickering nanoemulsions, or other nano-sized carriers in which the essential oil itself is encapsulated or dispersed at the nanoscale.
▪ Studies investigating applications in food systems, food preservation, active food packaging, edible films, edible coatings, postharvest preservation, or agricultural/plant protection systems, including biopesticide applications.
▪ Studies reporting at least one biological outcome and at least one safety outcome. Biological outcomes included antimicrobial, antibacterial, antifungal, antibiofilm, antioxidant, insecticidal, acaricidal, nematicidal, herbicidal, antigenotoxic, anti-inflammatory (when relevant to the intended application), or other bioactivity-related parameters. Safety outcomes included cytotoxicity, phytotoxicity, ecotoxicity, environmental toxicity, or other relevant safety assessments.
▪ Studies providing physicochemical characterization of the nanoformulation, including at least one of the following: particle size, polydispersity index, zeta potential, encapsulation efficiency and/or loading capacity, morphology, stability, release profile, or controlled-release properties.
▪ Studies in which nanoencapsulated EOs were incorporated into films, edible films, coatings, edible coatings, hydrogels, nanofibers, packaging materials, or other delivery matrices, provided that the essential oil was nanoencapsulated prior to incorporation.
▪ Articles published in peer-reviewed journals in any language.

#### 2.2.2. Exclusion Criteria

Studies were excluded if they met one or more of the following criteria:

▪ Review articles, systematic reviews, meta-analyses, conference abstracts, editorials, book chapters, patents, theses, or other non-original publications.
▪ Studies evaluating free (non-encapsulated) EOs or conventional formulations without nanoencapsulation.
▪ Studies in which nanoparticles were synthesized using EOs or plant extracts without nanoencapsulation of the essential oil itself (e.g., metal or metal oxide nanoparticles such as Ag, Au, ZnO, TiO_2_).
▪ Studies that did not evaluate the biological activity and/or safety outcomes of the essential oil nanoformulation.
▪ Studies that did not report any physicochemical characterization of the nanoformulation.
▪ Studies in which the essential oil was added directly to a film, coating, or packaging material without prior nanoencapsulation.
▪ Studies unrelated to food, postharvest preservation, packaging, or agricultural/plant protection applications.

### 2.3. Information Sources

A systematic literature search was conducted in the following databases: PubMed; Scopus and Web of Science. Additionally, the reference lists of included studies and relevant reviews were manually screened to identify additional eligible records not retrieved through the database search.

### 2.4. Search Strategy

A comprehensive search strategy was developed to identify studies investigating the application of nanoformulated EOs in food preservation, food safety, and agricultural protection. The search strategy combined controlled vocabulary terms (where available) with free-text keywords related to EOs, nanoencapsulation technologies, and their biological or technological applications.

The search terms were structured around three main concepts: (1) EOs and plant-derived bioactive compounds; (2) nanoformulation and delivery systems; and (3) food, agricultural, and preservation applications. The main keywords included combinations of the following:

- Essential oils and plant-derived compounds: “essential oil*” OR “volatile oil*” OR phytochemical* OR “plant extract*”.
- Nanoformulation technologies: nanoencapsulation OR nanoemulsion* OR nanocarrier* OR nanoparticle* OR nanocapsule* OR liposome* OR “solid lipid nanoparticle*” OR SLN* OR “nanostructured lipid carrier*” OR NLC*.
- Application and outcome domains: food OR “food preservation” OR “food safety” OR “food packaging” OR plant* OR agriculture OR “crop protection” OR postharvest OR biopesticide*.

Boolean operators (AND and OR) combined the search concepts to optimize retrieval of relevant studies. The search strategy was adapted for each electronic database according to its specific indexing system, search interface, and available search fields. Table 1 presents the complete search strategies used for PubMed, Scopus, and Web of Science.

Studies published between January 2016 and the final search date in 2026 were considered eligible to capture recent advances in nanoformulation technologies and the application of EOs in food preservation and agricultural systems. Only original research articles were considered eligible. Additional studies were identified through manual screening of the reference lists of included articles and relevant reviews.

### 2.5. Study Selection Process

All identified records were exported to Rayyan [17], and duplicates were removed. Two independent reviewers (SDG and LRS) performed:

- Title and abstract screening to exclude irrelevant studies.
- Full-text screening to assess eligibility based on inclusion/exclusion criteria.

**Figure 1:**
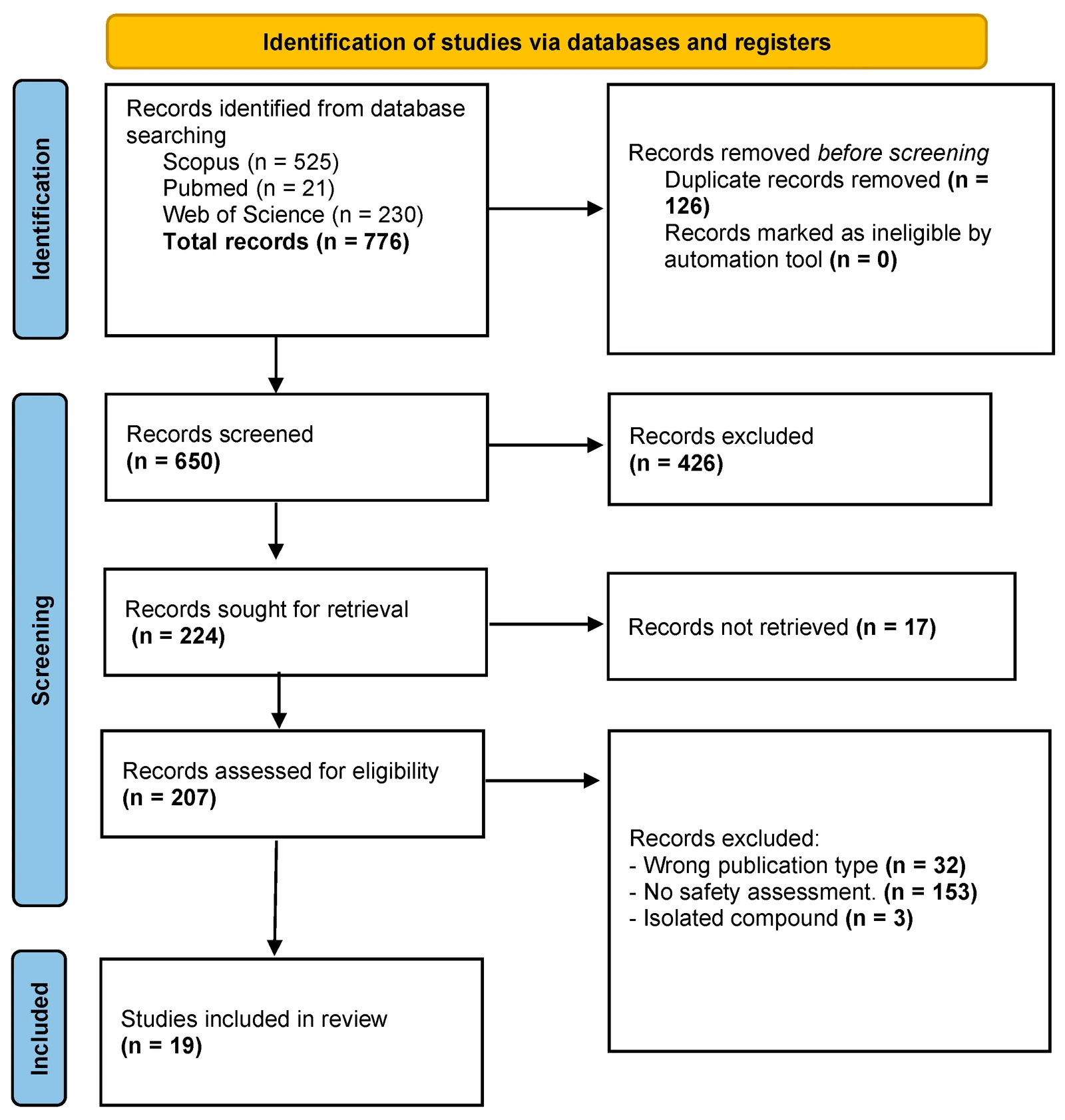
PRISMA Preferred Reporting Items for Systematic Reviews and Meta-Analyses) flow diagram illustrating the identification, screening, eligibility assessment, and inclusion process of studies examining the safety profile of the investigated compounds. Studies were excluded under “wrong publication type/study design” when they were non-original articles (e.g., reviews, editorials, commentaries), did not present empirical data, were not conducted in human participants, or did not provide relevant information regarding safety outcomes. Additional exclusions were applied to studies that did not assess safety-related parameters, did not evaluate the intervention of interest, investigated isolated compounds rather than the complete formulation/extract, or lacked sufficient information for data extraction. The final review included 19 studies that fulfilled all predefined eligibility criteria.

Disagreements were resolved by discussion. A PRISMA flow diagram documents the study selection process (Figure 1).

### 2.6. Data Collection Process

Two reviewers (SDG and LRS) extracted data independently using a standardized data extraction form aligned with the review’s objectives and piloted on a subset of included studies to ensure consistency and completeness. Reviewers resolved disagreements during extraction through discussion and consensus.

The following data were extracted from each included study:

Study characteristics:

- First author, publication year, country, and study design;
- Type of essential oil investigated;
- Nanoformulation strategy and characteristics (e.g., nanoemulsion, nanoparticles, liposomes, nanocapsules, carrier materials, particle size, encapsulation efficiency, and release properties).

Intervention and application characteristics:

- Essential oil formulation and treatment conditions;
- Comparison with non-encapsulated/free essential oil, when available;
- Application context (food preservation, active packaging, postharvest protection, or agricultural use).

Biological activity and outcome measures:

- Target microorganisms, pests, or biological models;
- Antimicrobial, antifungal, antioxidant, insecticidal, or preservation outcomes;
- Methods used for efficacy assessment and reported effect measures.

Safety and technological characteristics:

- Safety assessment methods (e.g., cytotoxicity, phytotoxicity, sensory evaluation, toxicity assays, and migration studies);
- Evidence of improved efficacy compared with free essential oil;
- Technological challenges, limitations, and authors’ main conclusions.

Any missing, incomplete, or unclear information identified during data extraction was recorded and, when necessary, attempts were made to contact the corresponding authors for clarification or additional data. The extracted information was subsequently cross-checked between reviewers before synthesis and analysis.

### 2.7. Data Items

#### 2.7.1. Outcomes

This review focused on the biological efficacy, technological performance, and safety of nanoformulated EOs. The following outcome domains were extracted from the included studies:

- Antimicrobial activity: antibacterial, antifungal, antibiofilm, and pathogen inhibition outcomes, including effects against foodborne microorganisms, plant pathogens, and spoilage organisms.
- Antioxidant activity: reported antioxidant capacity assessed through validated methods (e.g., DPPH, ABTS, FRAP assays) and related oxidative stability outcomes.
- Preservation outcomes: effects on food quality and shelf-life parameters, including microbial growth, weight loss, texture, colour, sensory characteristics, physicochemical properties, and deterioration indicators.
- Agricultural and pest-control outcomes: efficacy against plant pathogens, insects, or other agricultural targets.
- Nanoformulation performance outcomes: physicochemical characteristics, including particle size, polydispersity index, zeta potential, encapsulation efficiency, loading capacity, stability, and release behaviour.
- Safety outcomes: cytotoxicity, phytotoxicity, sensory evaluation, toxicity toward non-target organisms, migration assessment, and other reported safety indicators.

All reported outcome measures compatible with these domains were extracted, including quantitative results, comparative analyses with free/non-encapsulated EOs (when available), and qualitative assessments of improved efficacy.

#### 2.7.2. Other Variables

Additional variables extracted from each included study included:

- Study characteristics: first author, publication year, country, study design, and experimental setting.
- Essential oil characteristics: botanical source, plant species, chemical composition (when reported), and formulation type.
- Nanoformulation characteristics: encapsulation method, carrier materials, formulation parameters, and technological properties.
- Application characteristics: treatment conditions, dosage/concentration, exposure duration, target application (food preservation, active packaging, postharvest protection, or agricultural use), and comparison groups.
- Biological model characteristics: target microorganisms, biological systems, food matrices, crops, or insect species evaluated.
- Methodological variables: analytical methods used for efficacy and safety assessment, statistical approaches, and reported effect measures.

Any assumptions made regarding missing, incomplete, or unclear information were documented during data extraction. When relevant data were unavailable, the corresponding authors were considered for contact to request additional clarification or unpublished information.

### 2.8. Risk of Bias and Methodological Quality

The methodological quality and risk of bias of the included in vitro experimental studies were independently assessed by two reviewers (SDG and LRS) using the QUIN (Quality Assessment Tool for In Vitro Studies) [18]. This tool was selected because it is specifically designed to evaluate the methodological rigor, transparency, and reproducibility of in vitro research, including studies involving cell-based models, biomaterials, and laboratory-controlled experimental interventions.

The assessment was performed according to the following QUIN domains:

- S1: Clearly stated aims and objectives—evaluation of whether the study objectives and research hypotheses were explicitly described.
- S2: Detailed explanation of sample size calculation—assessment of whether the number of experimental replicates or samples was justified through an appropriate sample size calculation.
- S3: Detailed explanation of sampling techniques—evaluation of whether the selection, preparation, and handling of biological samples or experimental materials were adequately described.
- S4: Detailed description of experimental conditions—assessment of the completeness of methodological information, including protocols, culture conditions, treatment parameters, controls, and experimental procedures.
- S5: Randomization—evaluation of whether random allocation of samples, experimental units, or treatment conditions was applied.
- S6: Allocation concealment—assessment of whether allocation procedures were adequately concealed to minimize selection bias.
- S7: Blinding of investigators/outcome assessors—evaluation of whether investigators and/or assessors were blinded to treatment groups during experimental procedures and outcome evaluation.
- S8: Outcome measurement reliability—assessment of whether outcome measurements were performed using validated methods, calibrated instruments, standardized protocols, and appropriate replication.
- S9: Appropriate statistical analysis—evaluation of whether statistical methods were suitable for the study design, including appropriate tests, reporting of variability, and consideration of significance thresholds.
- S10: Reporting of results—assessment of whether results were clearly, completely, and transparently reported, including presentation of numerical data, statistical findings, and relevant experimental details.
- S11: Conclusions supported by data—evaluation of whether the experimental findings appropriately supported the conclusions and whether interpretations were consistent with the reported results.

Each domain was rated according to the QUIN criteria and categorized as low, high, or unclear risk of bias. When insufficient information was available to make a definitive judgement, the domain was classified as unclear risk of bias rather than assuming methodological inadequacy.

Two reviewers performed the assessments independently and resolved disagreements through discussion and consensus. The overall methodological quality profile was considered when interpreting the findings and evaluating the strength and reliability of the evidence derived from the included in vitro studies.

### 2.9. Effect Measures

Because substantial methodological and clinical heterogeneity existed across the included experimental studies, no common effect measure was prespecified or calculated for quantitative pooling. The review therefore used the effect measures reported by the individual studies to describe the direction and magnitude of effects within each outcome domain. These included percentage inhibition or reduction for antimicrobial, antifungal, insecticidal, and preservation outcomes; minimum inhibitory concentration (MIC), minimum bactericidal concentration (MBC), and minimum fungicidal concentration (MFC); mortality and dose–response measures, including LD50 and LD90; percentage radical-scavenging activity for antioxidant outcomes; percentage changes or differences in preservation and quality parameters; and quantitative physicochemical measures such as particle size, encapsulation efficiency, loading capacity, stability, and cumulative release.

Where studies directly compared nanoencapsulated and free/non-encapsulated essential oils, the reported comparative values were extracted and presented as reported by the original studies. No risk ratios, odds ratios, mean differences, standardized mean differences, or pooled effect estimates were calculated because the substantial heterogeneity in interventions, experimental models, outcome definitions, measurement methods, and reporting formats prevented meaningful quantitative synthesis.

The synthesis therefore focused on the reported direction and magnitude of effects, including differences in antimicrobial and antifungal activity, antioxidant capacity, preservation outcomes, safety profiles, and technological performance.

### 2.10. Synthesis Methods

#### 2.10.1. Criteria for Study Grouping

Studies were categorized according to predefined criteria to facilitate comparison and interpretation of findings. The included studies were grouped based on the following:

- Type of nanoformulated essential oil system: including nanoemulsions, polymeric nanoparticles, nanocapsules, liposomes, Pickering emulsions, and other nano-delivery platforms;
- Type of essential oil investigated: botanical source and chemical origin of the essential oil;
- Application context: food preservation, active packaging, postharvest protection, agricultural applications, or other biological applications;
- Outcome category: antimicrobial activity, antioxidant activity, preservation efficacy, safety assessment, and technological performance of the nanoformulation;
- Experimental model: in vitro laboratory assays, ex vivo/food-model studies, and in vivo or applied biological systems.

#### 2.10.2. Data Preparation

Data extracted from the included studies were prepared for narrative presentation by retaining the outcome measures and numerical results as reported in the original publications. Because essential oil types, nanoformulation technologies, experimental models, outcome definitions, and measurement methods varied substantially, no transformations were performed to convert results to a common statistical metric.

Where studies reported comparative outcomes between nanoencapsulated and free/non-encapsulated essential oils, the reported values were extracted and presented in their original units or scales. Percentage-based outcomes, including inhibition, reduction, mortality, antioxidant activity, and release, were retained as percentages. Physicochemical characteristics, including particle size, encapsulation efficiency, loading capacity, and related formulation parameters, were retained using the units reported by the individual studies.

Missing summary statistics were not imputed. When studies did not report a particular outcome, comparison, or quantitative parameter, these data were recorded as not reported. No assumptions or calculations were made to derive unreported effect estimates. This approach preserved the original study findings and avoided introducing additional uncertainty into the narrative synthesis.

#### 2.10.3. Presentation of the Results

The characteristics and findings of the included studies were presented in structured tables to facilitate cross-study comparison. Tables summarized study design and experimental model, essential oil characteristics, nanoformulation technology and physicochemical properties, intervention or application conditions, comparator characteristics, outcome domains, reported effect measures, and main findings.

Quantitative results were presented using the original units and measurement scales reported by each study. Where available, comparisons between nanoencapsulated and free/non-encapsulated essential oils were presented to facilitate assessment of differences in biological efficacy, technological performance, and safety.

The results of the individual studies were synthesized narratively and organized according to essential oil type, nanoformulation technology, experimental model, and outcome domain. Given the substantial heterogeneity among studies, no pooled quantitative estimates or meta-analytic forest plots were generated. The narrative synthesis therefore emphasized the direction and magnitude of reported effects, while the tabulated results provided the numerical findings and relevant methodological characteristics of each study.

#### 2.10.4. Synthesis Approach

A narrative synthesis was performed because the included studies showed substantial heterogeneity, including differences in nanoformulation strategies, essential oil sources, experimental models, application contexts, outcome measures, and analytical methodologies. The diversity of interventions and reported outcomes prevented quantitative meta-analysis.

The synthesis followed predefined grouping criteria based on essential oil type, nanoformulation technology, experimental model, and outcome domain. The reported effects of nanoformulated EOs were compared in terms of the direction and magnitude of their biological activity, including improvements in antimicrobial efficacy, antioxidant capacity, preservation outcomes, stability, controlled-release properties, and safety profiles.

Comparisons between nanoformulated EOs and free/non-encapsulated EOs were extracted when available to assess evidence of enhanced efficacy attributable to nanoencapsulation. Methodological differences, including formulation characteristics, treatment conditions, target organisms, analytical methods, and experimental settings, were considered when interpreting variations between studies. Risk-of-bias assessments were incorporated into the interpretation of the evidence’s strength and reliability.

#### 2.10.5. Exploration of Heterogeneity

Heterogeneity among the included studies was explored qualitatively through structured comparison of study characteristics, intervention characteristics, experimental models, application contexts, and outcome measures. Particular attention was given to differences in essential oil botanical source and chemical composition, nanoencapsulation strategy and carrier material, physicochemical characteristics of the nanoformulations, treatment conditions and concentrations, target microorganisms or biological models, and methods used to assess biological efficacy and safety. Studies were also compared according to food preservation, active packaging, postharvest, and agricultural applications.

Because of substantial methodological and clinical diversity among the included studies and the lack of sufficiently comparable quantitative outcome data, we did not perform statistical assessment of heterogeneity, subgroup meta-analysis, or meta-regression. Instead, potential sources of variation in findings were examined through predefined evidence-grouping categories and narrative comparison of the direction and magnitude of reported effects. Comparisons between nanoformulated and free/non-encapsulated essential oils were considered where available. Risk-of-bias assessments were also considered when interpreting differences in findings and the consistency of the evidence.

#### 2.10.6. Sensitivity Analysis

No formal statistical sensitivity analysis was performed because the substantial methodological and outcome heterogeneity among the included studies precluded quantitative meta-analysis. The robustness of the synthesized findings was instead assessed qualitatively by considering the consistency of results across different essential oil sources, nanoencapsulation strategies, experimental models, application contexts, and outcome domains. Particular attention was given to methodological limitations and risk-of-bias assessments when interpreting the strength and consistency of the evidence. Findings were also considered in relation to the availability of direct comparisons between nanoformulated and free/non-encapsulated essential oils.

#### 2.10.7. Reporting Risk of Bias Assessment

The risk of bias due to missing results arising from selective or incomplete reporting was assessed qualitatively. During data extraction and synthesis, attention was given to the completeness and consistency of reporting of formulation characteristics, release kinetics, biological outcomes, and safety assessments. Potential reporting bias was considered when interpreting differences in findings and the strength of the available evidence. Publication bias could not be formally assessed because of the small number of included studies and the absence of quantitative meta-analysis. The possibility that studies with positive findings were more likely to be published was therefore acknowledged as a potential source of bias.

#### 2.10.8. Certainty of Evidence

No formal certainty-of-evidence assessment, such as GRADE, was performed because the substantial methodological and outcome heterogeneity among the included studies precluded quantitative synthesis. Instead, confidence in the body of evidence was considered qualitatively by taking into account the consistency of findings across studies, methodological limitations, risk-of-bias assessments, completeness of reporting, and the characteristics of the experimental models used. Particular consideration was given to the predominantly laboratory-based and short-term nature of the available safety evidence, as well as the limited standardization of safety assessment protocols and incomplete reporting of relevant methodological and formulation characteristics. These factors were considered when interpreting the strength, consistency, and applicability of the overall evidence.

#### 2.10.9. Use of Tools

Rayyan supported record management, study screening, and organization of the review process [17]. Reviewers made inclusion, exclusion, and data extraction decisions independently.

## 3. Results

Database searches identified 776 records, including Scopus (*n* = 525), PubMed (*n* = 21), and Web of Science (*n* = 230). After removal of 126 duplicate records, 650 records were screened based on title and abstract, of which 426 were excluded because they did not meet the predefined eligibility criteria. The remaining 224 records were sought for retrieval; however, 17 articles could not be retrieved despite proactive efforts. Consequently, 207 full-text articles were assessed for eligibility. Following full-text assessment, 188 articles were excluded for the following reasons: wrong publication type (*n* = 32), absence of a safety assessment (*n* = 153), and studies investigating isolated compounds rather than essential oil formulations (*n* = 3). Ultimately, 19 studies met all eligibility criteria and were included in the systematic review.

The PRISMA flow diagram (Figure 1) presents the complete study identification and selection process, summarizing the numbers of records identified, screened, assessed for eligibility, and included in the final review.

### 3.1. Study Characteristics

The 19 studies included in this systematic review were published between 2019 and 2026 and investigated nanoformulated EOs for food preservation, agricultural protection, and antimicrobial applications. All studies employed experimental designs, predominantly laboratory-based approaches, including in vitro antimicrobial assays, ex vivo food models, and in vivo or postharvest preservation experiments. The studies spanned several geographical regions, with the most originating from China (*n* = 8), followed by India (*n* = 3), Brazil (*n* = 2), and Italy (*n* = 2). One study each was conducted in Iran, Algeria, and Spain.

The investigated EOs comprised a broad range of botanical sources, including *Origanum vulgare*, *Origanum majorana*, Thyme (*Thymus vulgaris*), Clove (*Syzygium aromaticum*), Cinnamon, Garlic (*Allium sativum*), *Coriandrum sativum*, Rose, Laurel, *Litsea cubeba*, *Melaleuca cajuputi*, *Monarda citriodora*, *Baccharis dracunculifolia*, *Eryngium campestre*, and combined essential oil formulations. Nanoformulation strategies varied considerably among studies and included nanoemulsions, polymeric nanoparticles, nanocapsules, liposomes, Pickering emulsions, dendritic mesoporous silica nanoparticles, and chitosan-based nano-delivery systems. Common encapsulation materials included chitosan, alginate, lipids, cellulose nanocrystals, Eudragit^®^ polymers, konjac glucomannan, and silica-based carriers.

The included studies widely reported nanoformulation characterization across key physicochemical parameters, including particle size, polydispersity index (PDI), zeta potential, morphology, encapsulation efficiency, and loading capacity. Most formulations exhibited nanoscale particle sizes, typically ranging from 25 to 260 nm, with several studies reporting narrow particle size distributions, high encapsulation efficiencies, and good colloidal stability. Researchers used a broad range of analytical techniques to characterize the formulations, including dynamic light scattering (DLS), scanning electron microscopy (SEM), transmission electron microscopy (TEM), Fourier-transform infrared spectroscopy (FTIR), X-ray diffraction (XRD), and thermogravimetric analysis (TGA). Chromatographic techniques were additionally used to characterize the chemical composition of the EOs and, in some cases, to assess changes in their composition following nanoencapsulation.

Regarding release behaviour, approximately half of the included studies investigated EO retention and/or release characteristics. Where release kinetics were experimentally assessed, nanoformulations generally exhibited an initial burst release followed by a more sustained, diffusion-controlled release phase, particularly in chitosan-based nanoparticles, liposomal systems, and polymeric matrices. However, several studies inferred controlled or sustained release based solely on indicators such as enhanced formulation stability, prolonged biological activity, or reduced volatility, without conducting formal release studies or kinetic modelling. This methodological limitation should be considered when interpreting claims of controlled release, as improved stability or prolonged bioactivity does not necessarily demonstrate a specific release mechanism or kinetic profile.

The biological activities evaluated were mainly antimicrobial, antifungal, antioxidant, insecticidal, and preservation-related outcomes. Microbial targets included foodborne bacteria such as *Escherichia coli* and *Staphylococcus aureus*, as well as plant and postharvest fungal pathogens including *Botrytis cinerea*, *Penicillium* spp., *Colletotrichum* spp., *Fusarium oxysporum*, and *Aspergillus* spp. Several studies investigated additional mechanisms, including membrane disruption, reactive oxygen species generation, inhibition of biofilm formation, inhibition of ergosterol biosynthesis, and suppression of mycotoxin production.

Most studies investigated nanoformulated EOs in food preservation systems, particularly edible coatings and active packaging applied to fruits such as strawberries, citrus fruits, apples, litchi, and apricots, as well as vegetables, mushrooms, rice, meat products, and other food matrices. Agricultural applications primarily involved botanical biopesticides and nano-enabled antifungal treatments targeting crop pathogens and insect pests. Across food preservation studies, the most frequently reported outcomes included inhibited microbial growth, delayed fungal spoilage, reduced weight loss, maintained firmness and sensory quality, preserved antioxidant compounds, and extended shelf-life compared with untreated controls. Overall, these findings indicate that nanoformulation can enhance the practical effectiveness of EOs in food and agricultural applications by improving their persistence and facilitating their sustained activity within the target system.

Most recent studies reported safety assessments, although methodologies and evaluation depth varied considerably. The reported approaches included cytotoxicity assays using mammalian cell lines, hemolysis assays, phytotoxicity testing, seed germination studies, sensory evaluation, toxicity assessments involving beneficial organisms, and investigations of nanoparticle migration into food matrices. Although most nanoformulations exhibited favourable preliminary safety profiles under the experimental conditions tested, the available evidence remains insufficient to establish their long-term safety. Several studies highlighted the need for further investigation of chronic toxicity, environmental impacts and fate, migration behaviour, regulatory compliance, and safety following industrial-scale production and application. Overall, the limited harmonization of safety assessment protocols and the scarcity of long-term and real-world exposure data remain important barriers to the broader translation of EO nanoformulations into food and agricultural applications.

Overall, the included studies suggest that nanoencapsulation can enhance the technological applicability of EOs by improving their physicochemical stability, aqueous dispersibility, bioavailability, controlled release, and persistence of biological activity compared with conventional EO formulations. However, studies did not consistently compare nanoencapsulated and free EOs directly, and the magnitude and consistency of any efficacy gains varied substantially across studies. This lack of standardized comparative assessment is an important limitation of the current evidence base and makes it difficult to determine the extent to which the observed improvements can be attributed specifically to nanoencapsulation.

Table 2 summarizes the main characteristics of the included studies, including publication year, country, study design, essential oil investigated, nanoformulation strategy, physicochemical characteristics, biological activity, application context, safety assessment, and main conclusions.

Overall, the included studies reported encouraging preliminary safety profiles for nanoencapsulated EOs, although the scope, endpoints, and methodologies used for safety assessment varied considerably. The most frequently evaluated outcomes included cytotoxicity, hemolysis, phytotoxicity, sensory acceptability, nanoparticle migration, and ecotoxicity. Under the experimental conditions investigated, most nanoformulations showed low acute toxicity and favourable preliminary compatibility with mammalian cells, food matrices, or plant systems, particularly when applied at optimized concentrations. However, safety assessments largely relied on short-term in vitro or laboratory-scale assays, with limited evaluation of dose–response relationships, repeated exposure, chronic toxicity, biodistribution, persistence, environmental fate, and potential interactions with non-target organisms. Moreover, the lack of standardized safety protocols and harmonized exposure conditions limits direct comparison across studies and constrains the generalizability of the available evidence. Comprehensive toxicological studies, realistic exposure scenarios, and regulatory-oriented risk assessments are therefore required to establish the long-term safety and environmental compatibility of nanoencapsulated EOs. Table 3 presents a synthesis of the safety outcomes reported across the included studies.

The technological performance of nanoencapsulated EOs was evaluated through physicochemical characterization, formulation stability, encapsulation efficiency, release behavior, and preservation performance. Despite the diversity of nanoencapsulation strategies, the included studies consistently demonstrated that nanoformulations improved the stability and applicability of EOs compared with conventional formulations. The most frequently reported technological advantages included reduced particle size, improved colloidal stability, enhanced protection against environmental degradation, controlled or sustained release, and increased biological persistence. Nevertheless, important challenges remain regarding formulation optimization, large-scale manufacturing, long-term storage stability, production costs, and regulatory approval. Table 4 presents a synthesis of the main technological outcomes reported across the included studies.

### 3.2. Risk of Bias and Methodological Quality

The methodological quality of the 19 included studies was assessed using an 11-item quality assessment checklist. Overall scores ranged from 50.0% to 72.7%, indicating predominantly moderate methodological quality. Eighteen studies (94.7%) were classified as having moderate methodological quality (50–70%), whereas only one study (5.3%) achieved a low risk/high methodological quality classification (>70%). Most studies clearly reported their objectives, experimental conditions, outcome measurements, results, and conclusions. However, sample size calculations and allocation concealment were consistently absent, while randomization and blinding procedures were infrequently reported, representing the main methodological limitations across the included studies (Table 5).

### 3.3. Reporting Bias

Reporting bias was assessed by examining the completeness and transparency of reporting across the included studies. Particular attention was given to the description of study objectives, experimental design, sample size calculation, randomization procedures, allocation concealment, blinding, outcome assessment, statistical analyses, and reporting of results and conclusions. The methodological quality of each study was evaluated using an 11-item quality assessment checklist, with individual items scored as 0 (not reported), 1 (partially reported), or 2 (adequately reported). An overall percentage score was subsequently calculated and used to classify studies by methodological quality.

Overall, the included studies demonstrated moderate reporting quality. Key elements, including study objectives, experimental conditions, outcome measures, statistical analyses, results, and conclusions, were generally described clearly. However, several methodological details relevant to assessing the robustness of the study designs and the potential for reporting bias were frequently insufficiently reported. None of the included studies reported a formal sample size calculation or allocation concealment, while randomization procedures and blinding of investigators or outcome assessors were inconsistently described or insufficiently detailed. These reporting limitations hinder a comprehensive assessment of methodological rigor and internal validity and may increase uncertainty regarding the risk of bias across the available evidence. Overall, the findings indicate moderate reporting quality, with substantial scope for improved transparency and standardized reporting of experimental design and methodological procedures.

### 3.4. Certainty of Evidence

The certainty of the evidence was assessed by considering the overall methodological quality, consistency of findings, and potential sources of bias across the included studies. Although most studies consistently reported beneficial effects of the investigated interventions, the overall certainty of the evidence was judged to be moderate. Methodological limitations, including the absence of prospective sample size calculations, limited reporting of randomization procedures, lack of allocation concealment, and infrequent blinding of investigators or outcome assessors primarily influenced this assessment.

Despite these limitations, the included studies generally employed relevant and clearly defined experimental models, provided clear descriptions of intervention protocols and outcome measures, and applied appropriate statistical methods. Moreover, the direction of the reported effects was broadly consistent across studies, supporting the overall robustness of the observed findings. Nevertheless, incomplete reporting of key methodological safeguards introduces uncertainty regarding the internal validity of the studies and limits confidence in the strength of the available evidence.

Accordingly, although the current evidence suggests that the investigated nanoformulations may exert beneficial biological effects across laboratory assays, food-model systems, postharvest applications, and agricultural/plant protection settings, the findings should be interpreted with some caution. Further well-designed experimental studies incorporating rigorous randomization, appropriate allocation concealment, blinding of investigators and outcome assessors, and prospective sample size calculations are warranted to strengthen the methodological quality of the evidence, increase certainty, and improve its translational relevance.

## 4. Discussion

This systematic review shows that nanoencapsulation has emerged as an effective strategy to overcome many intrinsic limitations of EOs, including high volatility, poor aqueous solubility, chemical instability, and rapid degradation under environmental conditions. Across the 19 included studies, nanoformulated EOs consistently exhibited enhanced physicochemical stability, improved dispersion in aqueous systems, and prolonged biological activity, supporting previous evidence that nanoscale delivery systems can substantially improve the practical applicability of plant-derived bioactive compounds.

The predominance of nanoemulsions, chitosan-based nanoparticles, liposomes, polymeric nanocarriers, and Pickering emulsions reflects current trends in nanotechnology for food preservation and sustainable agriculture [38,39,40]. As in previous reviews, the findings indicate that these delivery systems not only protect volatile EO constituents from oxidation and evaporation but also enable more efficient interaction with microbial cells by increasing surface area and improving bioavailability [41,42]. Eight of the nineteen included studies (42.1%) experimentally demonstrated sustained or controlled-release behaviour through release kinetics or diffusion analyses, whereas several additional studies inferred prolonged release from enhanced stability or extended antimicrobial activity. Controlled release was consistently associated with prolonged biological activity and reduced volatility of the encapsulated essential oils [43].

The biological outcomes reported across the included studies were remarkably consistent. Nanoencapsulated EOs generally exhibited stronger antimicrobial and antifungal activity than their conventional counterparts, particularly against foodborne pathogens such as *Escherichia coli* and *Staphylococcus aureus* and postharvest fungi including *Botrytis cinerea*, *Penicillium* spp., and *Fusarium oxysporum*. These observations consistent with previous reports suggesting that nanoencapsulation can enhance the antimicrobial performance of essential oils by improving their availability at microbial interfaces, facilitating interactions with microbial cell membranes, enabling prolonged release, and reducing the interference of food constituents with antimicrobial activity [43]. Importantly, enhanced antimicrobial efficacy often translated into improved preservation outcomes, including delayed microbial spoilage, reduced weight loss, maintained firmness, preserved antioxidant compounds, and extended shelf life in fruits, vegetables, meat products, and cereals [44].

A key finding of this review is that nanoencapsulation may also improve the safety profile of EO-based formulations. Most studies reported low cytotoxicity, minimal hemolytic activity, acceptable phytocompatibility, or no significant nanoparticle migration under the tested conditions [20,22,23,24,25,26,27,28,30,32,33]. In several cases, nanoencapsulation reduced the phytotoxic effects observed with free EOs while maintaining, or even improving, antimicrobial efficacy [20,24,25]. These findings support the hypothesis that controlled delivery systems reduce local exposure peaks and improve the distribution of active compounds [45]. Nevertheless, the available safety evidence remains largely preliminary, as most investigations relied on short-term in vitro assays, whereas comprehensive evaluations of chronic toxicity, environmental fate, food migration, and human exposure remain scarce.

Despite these encouraging findings, the review also highlights several important limitations within the current body of evidence. Direct comparisons between nanoencapsulated and non-encapsulated EOs were not consistently performed, making it difficult to quantify the true magnitude of the technological advantage provided by nanoformulation [19,21,22,26,27,30,32,33,37]. In several studies, researchers evaluated nanoformulations primarily against formulation controls or conventional emulsions rather than the corresponding free EO, limiting the strength of conclusions about enhanced efficacy [22,27,30,32]. Likewise, controlled-release behaviour was often inferred from improved stability or prolonged biological activity rather than demonstrated through formal release kinetics [19,20,25,26,27,29,30,32,34,35,37]. Only a subset of studies quantitatively characterized EO release over time, using approaches such as dialysis or release models [21,22,24,28,31,33]. Considerable methodological heterogeneity across studies regarding EO sources, encapsulation materials, nanoparticle characteristics, antimicrobial assays, and preservation models further limits comparisons and precludes quantitative synthesis.

Overall, these findings reinforce the growing consensus that nanoencapsulation is one of the most promising approaches for translating the well-established biological activities of EOs into practical food preservation and agricultural applications. However, the current evidence also indicates that further standardized research is required to establish robust efficacy data, clarify long-term safety, and facilitate regulatory approval and industrial implementation.

### 4.1. Limitations of the Evidence

Despite the promising findings reported across the included studies, several methodological and translational challenges limit the overall strength of the evidence. First, all included studies employed experimental laboratory-based designs, with most relying on in vitro antimicrobial assays, ex vivo food models, or controlled postharvest experiments. None of the studies included clinical evaluations or large-scale industrial validation, limiting the generalizability of the findings to real-world food production and agricultural systems.

Considerable heterogeneity was observed among the included studies regarding the EOs investigated, nanoencapsulation technologies, encapsulating materials, formulation methods, particle characteristics, biological assays, and outcome measures. Nanoformulations included nanoemulsions, polymeric nanoparticles, liposomes, Pickering emulsions, mesoporous silica nanoparticles, and chitosan-based systems, making direct comparisons across studies difficult. Likewise, antimicrobial efficacy was assessed using different microorganisms, preservation models, concentrations, exposure times, and analytical methods, precluding quantitative synthesis or meaningful comparison of effect sizes.

Another important limitation concerns the inconsistency of comparative analyses. Although nanoencapsulation is generally proposed to enhance the stability and biological activity of EOs, several studies did not include direct comparisons with the corresponding free (non-encapsulated) EOs. In many cases, researchers inferred improved efficacy from enhanced physicochemical stability or prolonged activity rather than demonstrating it experimentally under identical conditions. Consequently, the superiority of nanoencapsulation over conventional formulations cannot be considered conclusive across all applications.

Evidence regarding the controlled-release behaviour of nanoencapsulated EOs was also limited and heterogeneous. Evidence regarding the controlled-release behaviour of nanoencapsulated EOs was limited and heterogeneous. Although approximately half of the included studies experimentally evaluated EO release profiles, several studies inferred sustained or controlled release from indirect indicators rather than formal release kinetics. Specifically, Molon et al. [20], Yu et al. [25], Baldassarre et al. [34], and Deepika et al. [36] suggested sustained or controlled release based on improved formulation stability, prolonged biological activity, mesoporous carrier structure, delayed EO availability, or other indirect observations, without conducting formal release kinetics analyses or modelling. This methodological limitation makes it difficult to establish whether the observed prolonged biological activity was attributable to controlled release and limits mechanistic understanding of the factors underlying the enhanced performance of nanoformulated EOs. It also complicates meaningful comparisons of release behaviour and delivery efficiency across different nanoformulation systems.

Safety assessment represented another major source of heterogeneity. Although most recent studies included preliminary evaluations such as cytotoxicity, hemolysis, phytotoxicity, seed germination, sensory analysis, or nanoparticle migration, these assessments were generally limited to short-term laboratory models. Comprehensive investigations addressing chronic toxicity, repeated exposure, biodistribution, environmental fate, ecotoxicity, nanoparticle accumulation, and interactions with complex food matrices remain scarce. Furthermore, studies lacked standardized safety evaluation protocols, making comparisons difficult.

Finally, several studies identified important technological and translational barriers that may hinder the practical implementation and commercial adoption of nanoencapsulated EOs. These included limited scalability of nanoformulation processes, insufficient assessment of production costs and economic feasibility, potential formulation instability during long-term storage, regulatory uncertainty, and the lack of industrial-scale pilot validation. In addition, the limited availability of standardized manufacturing and quality-control procedures may further constrain reproducibility and large-scale production. Collectively, these limitations indicate that, despite encouraging laboratory-scale evidence, substantial technological, economic, regulatory, and validation gaps remain. Further standardized, multidisciplinary, and application-oriented research is therefore required to establish the scalability, cost-effectiveness, long-term stability, safety, and regulatory feasibility of nanoencapsulated EOs before their widespread adoption in commercial food preservation and agricultural applications.

### 4.2. Limitations of the Review

This systematic review has several limitations to consider when interpreting its findings. First, although we used a comprehensive search strategy across three major electronic databases (Scopus, PubMed, and Web of Science), we may not have identified relevant studies indexed exclusively in other databases or available as grey literature (e.g., conference proceedings, dissertations, technical reports, and preprints). Consequently, publication bias cannot be ruled out, particularly because studies reporting positive findings are more likely to be published than those with negative or inconclusive results.

Second, the review included only peer-reviewed original research articles that reported both biological efficacy and a safety assessment. While this approach strengthened the focus on translational evidence, it also excluded numerous studies investigating nanoencapsulated EOs that did not assess safety outcomes. As a result, potentially relevant information regarding formulation development, antimicrobial mechanisms, or technological performance may not have been captured.

Third, including only studies that met predefined efficacy and safety criteria may have reduced the breadth of available evidence. This criterion was adopted to provide a more comprehensive assessment of the potential benefits and safety considerations of nanoencapsulated EOs; however, it may have excluded studies focused exclusively on formulation optimization or specific technological characteristics.

Fourth, the substantial methodological diversity of the included studies prevented quantitative pooling of the results. Consequently, this review relied on a qualitative synthesis rather than a meta-analysis. Although this approach allowed consideration of findings from diverse experimental models and nanoencapsulation systems, it limits the ability to estimate pooled effects or formally quantify differences in efficacy between formulations.

Finally, the rapidly evolving nature of nanoencapsulation technology represents an inherent limitation of the present review. New nanoformulation strategies, characterization approaches, safety data, and application studies continue to emerge. Therefore, the findings reflect the evidence available at the time of the literature search and may require updating as additional evidence becomes available.

### 4.3. Implications for Practice, Policy, and Future Research

The findings of this systematic review suggest that nanoencapsulation is a promising strategy for overcoming many intrinsic limitations of EOs, including poor water solubility, high volatility, rapid degradation, and limited stability. Across the included studies, nanoencapsulation generally enhanced physicochemical stability, improved dispersion, prolonged biological activity, and, in many cases, increased antimicrobial and antioxidant efficacy compared with conventional formulations. These properties support the potential application of nanoencapsulated EOs as natural alternatives to synthetic preservatives in food preservation and as environmentally friendly bioactive agents for crop protection. However, current evidence suggests that commercial implementation should proceed cautiously until further validation under industrial conditions is available.

From a practical perspective, nanoencapsulated EOs may help extend the shelf life of perishable foods, reduce postharvest losses, enhance active food packaging technologies, and decrease dependence on synthetic antimicrobial agents and conventional pesticides. Their ability to provide controlled or sustained release may also reduce application frequency while maintaining biological efficacy. Nevertheless, practical implementation requires optimizing formulation stability during storage, enabling reproducible large-scale manufacturing, developing cost-effective production processes, and ensuring compatibility with existing food processing and agricultural practices. Standardized quality control procedures and manufacturing protocols will be essential to ensure batch-to-batch consistency and product performance.

The review also has important implications for regulatory policy. Although most included studies reported favourable preliminary safety profiles, current evidence remains insufficient to support widespread regulatory approval of many nanoencapsulated essential oil formulations. Regulatory agencies will require comprehensive toxicological data addressing chronic exposure, nanoparticle migration into food, environmental persistence, ecotoxicity, biodegradability, and occupational exposure during manufacturing. Harmonized international guidelines for the characterization, safety assessment, labeling, and risk evaluation of nano-enabled food preservatives and agricultural products are needed to facilitate regulatory decision-making and promote consumer confidence. Developing standardized testing frameworks would also improve comparability across studies and accelerate translation from laboratory research to commercial applications.

The findings additionally highlight several priorities for future research. Well-designed studies should directly compare nanoencapsulated formulations with their corresponding free EOs using standardized experimental protocols to determine the true magnitude of performance improvements. Future work should place greater emphasis on comprehensive release kinetics, long-term physicochemical stability, interactions with complex food matrices, and the mechanisms underlying enhanced antimicrobial activity. Future investigations should also evaluate sensory properties, consumer acceptance, economic feasibility, and life-cycle sustainability, which remain poorly addressed despite their importance for industrial adoption.

Finally, more translational research is needed to bridge laboratory findings and real-world applications. Pilot-scale manufacturing studies, industrial validation trials, and long-term storage experiments under commercial conditions are necessary to establish the practical effectiveness of nanoencapsulated EOs. Similarly, field studies evaluating agricultural performance under diverse environmental conditions, along with standardized environmental risk assessments, will be essential to determine whether these technologies can provide sustainable alternatives to conventional chemical preservatives and pesticides. Collectively, addressing these knowledge gaps will strengthen the evidence base and support the responsible development of nanoencapsulated EOs as safe and effective technologies for food preservation and plant protection.

## 5. Conclusions

This systematic review indicates that nanoencapsulation can enhance the technological performance of EOs by improving their stability, dispersibility, release behaviour, and, in many cases, antimicrobial and antioxidant efficacy in food preservation and agricultural applications. Nanoformulated EOs therefore show considerable potential as sustainable alternatives to synthetic preservatives and pesticides, with possible benefits for shelf-life extension, postharvest quality, and environmentally sustainable crop protection. However, the evidence remains predominantly laboratory-based and is characterized by substantial heterogeneity in nanoformulation strategies, biological models, and safety assessment methods. Although preliminary safety findings are generally encouraging, evidence on long-term toxicity, environmental impacts, regulatory compliance, and industrial scalability remains limited. Future research should prioritize standardized methodologies, direct comparisons with free EOs, comprehensive safety assessment, and pilot- or commercial-scale validation to support the safe, effective, and sustainable translation of nanoencapsulated EOs into practical food and agricultural applications.

## Figures and Tables

**Table 1:** Electronic database search strategies.

Database	Seach String	Restrictions
Pubmed	((“essential oil*” OR “volatile oil*” OR phytochemical*) AND (nanoencapsulation OR nanoemulsion* OR nanocarrier* OR nanoparticle* OR nanocapsule* OR liposome* OR “solid lipid nanoparticle*” OR SLN* OR “nanostructured lipid carrier*” OR NLC*) AND (food OR “food preservation” OR “food safety” OR plant* OR agriculture OR “crop protection”))	2016–2026
Scopus	(“essential oil*”) AND (nanoencapsulation OR nanoemulsion* OR nanocapsule* OR liposome* OR “solid lipid nanoparticle*” OR “nanostructured lipid carrier*”) AND (“food preservation” OR “food packaging” OR “crop protection” OR postharvest OR biopesticide*)	2016–2026
Web of Science	((“essential oil*”) AND (nanoencapsulation OR nanoemulsion* OR liposome* OR nanocapsule* OR “solid lipid nanoparticle*” OR “nanostructured lipid carrier*”) AND (“food preservation” OR “food packaging” OR “crop protection” OR postharvest OR biopesticide*))	Open Acess Article 2016–2026

**Table 2:** Included studies (*n* = 19).

Study No. Year Country Study Design Reference	Essential Oil	Nanoformulation	Nanoformulation Characteristics	Release Properties	Comparison with Non-Encapsulated EO	Biological Activity	Target Microorganisms	Food/Agricultural Application	Treatment Conditions	Preservation Outcomes	Safety Assessment	Evidence of Improved Efficacy vs. Free EO	Technological Challenges Identified	Main Conclusion
1 2026 Iran Experimental study [19]	*Eryngium campestre*	EON incorporated into a sodium alginate edible coating.	Prepared using Tween 80 (surfactant), ethanol (co-surfactant), high-speed homogenization, and ultrasonication. Droplet size: 76–118 nm; PDI: 0.288–0.401; zeta potential: −31 to −24 mV; encapsulation efficiency: 65.25–82.55%.	Controlled release is expected from the nanoemulsion–alginate matrix; no direct release kinetics study was performed.	No direct comparison with free EO was performed. The authors state that nanoemulsification improves dispersion, stability, and bioavailability compared with free EO, based on the previous literature.	Antimicrobial and antioxidant activity. Reduced microbial growth, lipid oxidation, protein degradation, and quality deterioration during storage.	Total mesophilic bacteria, psychrotrophic bacteria, and Enterobacteriaceae.	Edible coating to preserve ostrich meat during refrigerated storage.	Meat samples were immersed in SA/EON coating solutions for 15 min, drained, vacuum-sealed, and stored at 4 ± 1 °C for 12 days.	SA/EON coatings reduced pH increase, free fatty acids, total volatile basic nitrogen, peroxide value, TBARS, microbial counts, and drip loss, and improved texture and sensory quality. The 5% EON formulation showed the strongest preservation effect.	A trained panel (*n* = 10) conducted the sensory evaluation. No adverse sensory effects observed. Alginate and EO are considered food-compatible; regulatory validation is still required.	Not experimentally demonstrated. Improvement attributed to nanoemulsion properties (smaller droplets, improved dispersion and interaction with microbial cells/lipids).	Laboratory-scale validation only; no industrial trials, migration studies, long-term consumer acceptance, or cost optimization.	A sodium alginate coating containing an *E. campestre* EO nanoemulsion effectively extended the refrigerated shelf life of ostrich meat by combining EO bioactivity with alginate barrier properties. The 5% EON treatment provided the best preservation performance.
2 2026 Brazil Experimental study [20]	*Origanum vulgare* L.	Eudragit^®^ S100 polymeric OVEO–NC. Prepared by interfacial deposition of a preformed polymer after solvent displacement.	Particle size (DLS): 229 ± 16 nm for optimized formulation (OVEO–NC–5). SEM diameter: 143 ± 26 nm. Zeta potential: −39.7 ± 0.8 mV. Encapsulation efficiency: 90.72 ± 2.10%. Loading capacity: 81.18 ± 1.75%. Morphology: spherical nanocapsules. FTIR confirmed interaction/incorporation of OVEO into the polymer matrix. TGA indicated improved thermal stability of encapsulated EO compared with free EO.	No direct release kinetics study was performed. The authors inferred potential sustained/controlled release based on improved stability, polymeric barrier properties, and prolonged antifungal activity.	Free Oregano EO was compared with OVEO–NC–5 in vitro and in vivo. Both formulations showed antifungal activity, but nanoencapsulation improved persistence and effectiveness, particularly at low concentrations and during postharvest storage.	Antifungal activity was evaluated by inhibiting conidial germination and suppressing postharvest fungal infection.	*Botrytis cinerea* *Colletotrichum fructicola* *Colletotrichum gloeosporioides* *Penicillium digitatum*	Postharvest preservation of apples (*Malus domestica* cv. Gala) as a biocontrol strategy against fungal spoilage (*C. gloeosporioides*). Agricultural plant protection/postharvest disease management application.	In vitro: Oregano EO and OVEO–NC–5 tested at 0.00625–0.4% (*v*/*v*) against fungal conidia; incubation at 25 °C for 48 h. In vivo: Apples inoculated with *C. gloeosporioides*; treatments with free Oregano EO or OVEO–NC–5 at 0.025 and 0.05% (*v*/*v*). Fruits were stored at 24 ± 1 °C and 95% relative humidity for 21 days. Lesion diameter was measured on days 7, 14, and 21.	OVEO–NC–5 significantly reduced fungal lesion development in apples compared with untreated controls. At 0.05% (*v*/*v*), nanoencapsulated Oregano EO completely inhibited visible symptoms caused by *C. gloeosporioides*. At 0.025%, lesion diameter was reduced by approximately 62% compared with infected untreated controls.	Limited phytotoxicity assessment. Free Oregano EO at higher concentrations caused tissue damage in apple fruit, whereas OVEO–NC–5 reduced phytotoxic effects while maintaining antifungal activity. No cytotoxicity, ecotoxicity, or environmental safety assays were performed.	Yes. Nanoencapsulation improved antifungal performance: Greater inhibition of conidial germination after 48 h at lower concentrations. Free Oregano EO showed reduced effectiveness over time, whereas OVEO–NC maintained inhibition. In apples, OVEO–NC reduced lesion development more effectively than free OVEO and prevented symptoms at 0.05%.	EOs have high volatility and limited stability. Direct application may cause phytotoxic effects on plant tissues. Nanoencapsulation is required to improve dispersion, protect bioactive compounds, and prolong activity. Release behavior requires further investigation under real postharvest conditions.	Eudragit^®^ S100 nanocapsules effectively encapsulated oregano essential oil, enhancing its antifungal activity and postharvest protection potential compared with free essential oil. Nanoencapsulation improved stability, reduced phytotoxicity, and enabled more effective control of fungal spoilage in apples, supporting its potential application as a natural postharvest preservative.
3 2026 China Experimental laboratory study (in vitro/ex vivo food model study) [21]	Clove	Clove NEO stabilized with carboxymethyl chitosan and Tween 80; incorporated into KC hydrogel films.	Nanoemulsion optimized with 3% Tween 80; particle size 67.0 ± 3.77 nm; PDI 0.31; zeta potential −36.02 mV, indicating good stability.	Controlled and sustained Clove EO release from films; Ritger–Peppas model best fit; Fickian diffusion mechanism (*n* = 0.21–0.33).	Not directly compared with free Clove EO; nanoencapsulation improved dispersion, stability, reduced volatilization, and enabled controlled release within the film matrix.	Antioxidant activity (DPPH/ABTS scavenging up to ~90%); antibacterial activity against *E. coli* and *S. aureus*.	*Escherichia coli* and *Staphylococcus aureus*	Active biodegradable packaging film for strawberry preservation.	Strawberries stored at 4 °C and 60% RH for 10 days; films tested at different Clove NEO concentrations (KC0.2–KC0.8).	KC0.6 film showed optimal performance: delayed mold growth, reduced weight loss (9.9%), maintained firmness and soluble solids, and outperformed the control and PE film in later storage stages.	Soil degradation test, seed germination assay, and cytotoxicity tests (HIEC-6 and Caco-2 cells). KC0.6 showed good biocompatibility; KC0.8 reduced cell viability.	Nanoemulsion formulation provided enhanced stability, sustained release, and prolonged antimicrobial/antioxidant activity, overcoming the expected limitations of free EO (volatility and poor water solubility).	Balancing Clove NEO concentration with mechanical properties, release rate, antimicrobial efficacy, and cytotoxicity; excessive loading caused structural weakening and reduced safety.	Clove NEO-loaded κ-carrageenan hydrogel films, especially KC0.6, provide a biodegradable, safe, and effective active packaging system that extends strawberry shelf life through controlled release of Clove EO and antimicrobial/antioxidant activity.
4 2025 China Experimental study [22]	Thyme	LIP-TEO-CuNPs; liposomes prepared using lecithin and cholesterol; subsequently incorporated into chitosan coating film	Particle size: ~190 nm; PDI: 0.284; zeta potential: −22.45 mV; morphology: spherical/unilamellar vesicles confirmed by TEM and SEM; FTIR and XPS confirmed incorporation of Thyme EO and CuNPs; good dispersion and stability	Controlled release of Thyme EO and CuNPs from liposomes. Thyme EO cumulative release: 38.99% after 12 h and 65.17% after 168 h (7 days). CuNP cumulative release: 13.91% after 12 h and 15.17% after 168 h	LIP-TEO was compared with LIP-TEO-CuNPs and blank liposomes. Direct comparison with free Thyme EO was not performed. Nanoformulation improved stability, controlled release, and antimicrobial performance through liposomal delivery.	Antioxidant activity (DPPH, FRAP, ABTS assays); antibacterial activity (~95% inhibition reported for LIP-TEO-CuNPs); antifungal activity (~50% inhibition of fungal growth); enhanced activity due to synergistic effects of Thyme EO and CuNPs	Bacteria: *Escherichia coli*, *Staphylococcus aureus*; *Fungi*: *Rhizopus*, *Colletotrichum*	Postharvest food preservation; chitosan-based edible coating applied to fresh litchi (*Litchi chinensis cv*. Guiwei)	Litchi fruits immersed in chitosan/liposome coating solutions for 5 min; stored at 20 °C and 75% relative humidity for 9 days; treatment groups included CS, CS/LIP-TEO, and CS/LIP-TEO-CuNPs (1–10% liposome concentrations)	Delayed browning and decay; reduced weight loss; maintained color, soluble solids, titratable acidity, vitamin C content; reduced membrane damage and polyphenol oxidase/peroxidase activity; improved phenolic/flavonoid retention; delayed volatile compound changes and maintained freshness	Cytotoxicity was evaluated using MTT assay and flow cytometry on fibroblasts and endothelial cells; copper accumulation in litchi flesh was measured by inductively coupled plasma optical emission spectrometer. Cell viability remained ~99%; no significant copper accumulation detected	Limited. The study did not directly compare free Thyme EO. However, nanoencapsulation provided controlled release and improved antimicrobial performance compared with non-Cu liposomal Thyme EO (LIP-TEO).	Additional complexity of liposome production; need to control CuNP release; potential concerns regarding nanoparticle migration; scalability and cost were not evaluated.	Green-synthesized CuNPs were successfully encapsulated in liposomes containing thyme EO. The LIP-TEO-CuNPs system showed controlled release, strong antioxidant and antimicrobial activity, and synergistic inhibition of bacteria and fungi. Incorporated into chitosan coatings, it effectively delayed litchi browning, microbial spoilage, and quality deterioration, and maintained fruit freshness during storage. Preliminary safety assessment showed low cytotoxicity and no significant copper accumulation in fruit.
5 2025 Algeria Experimental study [23]	*Origanum majorana* and *Foeniculum vulgare*	NEO of essential oils. Coarse emulsions were prepared using 4% essential oil with Tween 80 surfactant (1:5 *v*/*v*), followed by probe sonication.	NEO showed nanoscale particle size (~121 nm). Characterization included particle size distribution, PDI, zeta potential, stability, pH, density, and refractive index. Particle size: *O. majorana* NEO 121.26 ± 8.37 nm; *F. vulgare* NEO 121.26 ± 5.77 nm. PDI: 0.27 (*O. majorana*) and 0.31 (*F. vulgare*). Stability: 92.41% and 89.17%, respectively.	No explicit release kinetics or controlled-release study was reported. The authors evaluated stability and nanoscale dispersion, but did not provide an in vitro release profile.	Yes. The authors tested free EOs alongside nanoemulsions in antimicrobial, antifungal, and cytotoxicity experiments. The study compared EO and NEO activity against bacteria and fungi, and assessed their performance in fruit preservation.	Antimicrobial: antibacterial and antifungal activity was measured by MIC, MBC, MFC, disk diffusion, and fungal growth inhibition. Antioxidant-related fruit quality parameters, including ascorbic acid content and physicochemical preservation parameters, were monitored. Anticancer/cytotoxic activity: evaluated using the MTT assay.	Bacteria: *Escherichia coli*, *Salmonella typhimurium*, *Klebsiella pneumoniae*, *Pseudomonas aeruginosa*, *Staphylococcus aureus*, *Bacillus subtilis.* Fungi: *Aspergillus niger*, *A. flavus*, *Fusarium culmorum*, *Alternaria citri*, *Penicillium digitatum*, *P. expansum*, *P. citrinum*.	Postharvest food preservation. Nanoemulsions were applied as coatings on citrus fruits (orange and lemon) to control fungal spoilage and extend storage quality.	EO nanoemulsions were applied as coatings to inoculated citrus fruits. Lemon fruits were inoculated with Penicillium spores, coated, dried, and stored. Orange fruits coated with *O. majorana* nanoemulsion were evaluated after 21 days for quality and sensory characteristics.	Nanoemulsion coatings reduced fungal development on citrus fruits. Lemon fruits coated with aromatic oil nanoparticles resisted *Penicillium citrinum* infection, while fruits coated with crude oils showed fungal growth. Orange fruits treated with *O. majorana* nanoemulsion showed improved firmness, sensory scores, and preservation characteristics compared with controls.	Cytotoxicity was evaluated using the MTT assay on HeLa normal cells and HepG2 and HT29 cancer cell lines. Nanoemulsions showed dose-dependent effects on cell viability.	Partially yes. Nanoemulsions improved antifungal performance, especially *O. majorana* NEO, which achieved up to 96.55% reduction in *P. citrinum* mycelial growth compared with free oil. Nanoformulation enhanced preservation in fruit-coating applications. However, the antibacterial activity of nanoemulsions was not always superior to that of free EO; some assays showed weaker inhibition zones with NEO.	The study did not explicitly discuss technological challenges. Indirect challenges include variability between EOs, changes in volatile composition after nanoemulsification, differences in antimicrobial effectiveness between oils, and the need to optimize nanoemulsion stability and application performance.	Essential oil nanoemulsions of *O. majorana* and *F. vulgare* were successfully produced and characterized as nanoscale delivery systems. *O. majorana* nanoemulsion showed strong antifungal activity and potential as a natural coating for citrus preservation. Nanoemulsification improved EO functionality, particularly for postharvest fungal control, and offers a promising approach for food preservation applications.
6 2025 India Experimental study [24]	*Coriandrum sativum*	*Coriandrum sativum* EO-loaded chitosan nanoemulsion (Ne-CSEO). Prepared by ionic gelation using chitosan and sodium tripolyphosphate. Tween-20/Tween-80 were used as emulsifiers.	Particle size: 78.67 nm (DLS); SEM size range: 50.79–85.27 nm. Zeta potential: +31.37 mV. PDI measured. Encapsulation potency: 92.11% maximum. Loading potency: 5.04%. Encapsulation yield: 27.96%. FTIR confirmed interaction between *Coriandrum sativum* EO and chitosan; XRD showed reduced crystallinity after encapsulation.	In vitro *Coriandrum sativum* EO release was studied in ethanol/PBS (pH 7.0) for 7 days. Showed three phases: initial burst release (0–12 h), sustained diffusion phase (12–72 h), and slow release phase (72–168 h).	Direct comparison between free *Coriandrum sativum* EO and Ne-CSEO. Ne-CSEO showed stronger antifungal, anti-fumonisin, antioxidant, and preservation effects at lower concentrations.	Antifungal, anti-fumonisin, and antioxidant activities. Mechanisms were evaluated through ergosterol inhibition, ion leakage, membrane damage (PI staining), and molecular docking of linalool with the Fum1 protein.	Main target: *Fusarium proliferatum* strain FP-BRC-R2 (fumonisin producer). Also tested against *F. verticillioides*, *Aspergillus flavus*, *A. niger*, *A. repens*, *Penicillium italicum*, *Alternaria alternata*, *A. humicola*, and *Curvularia lunata*.	Stored rice preservation. Rice varieties were screened; the final storage experiment used Gobindobhog rice, treated by fumigation with *Coriandrum sativum* EO or Ne-CSEO in sealed containers.	In vitro: CSEO MIC = 0.7 µL/mL; Ne-CSEO MIC = 0.4 µL/mL. Storage: rice stored for 6 months at 25 ± 2 °C and 75% RH. Ne-CEO was applied at 0.4 µL/mL.	Ne-CSEO achieved 100% inhibition of *F. proliferatum* infestation and complete suppression of FB1 and FB2 fumonisin production after 6 months. Improved carbohydrate/protein retention, reduced lipid peroxidation, and maintained sensory properties.	Phytotoxicity assay: no negative effect on rice seed germination. Organoleptic evaluation with 10 participants. Rice quality parameters (carbohydrates, proteins, lipid oxidation) assessed.	Strong evidence: Ne-CSEO required a lower concentration (0.4 vs. 0.7 µL/mL), produced stronger antifungal activity, achieved complete fungal suppression in stored rice, improved antioxidant activity, and better maintained food quality.	Limited explicitly stated challenges. Relevant issues mentioned: EO instability, loss of oil components, insufficient polymer availability at high EO ratios, possible oil coalescence, and the need to optimize the chitosan/EO ratio.	Chitosan nanoencapsulation improved coriander EO stability, controlled release, antifungal and anti-fumonisin efficacy, making Ne-CSEO a promising natural fumigant for rice preservation and fungal/mycotoxin control.
7 2025 China Experimental study [25]	Thyme	TEO@DMSNs. DMSNs served as nanoscale carriers for loading EOs and for controlled delivery.	Dendritic mesoporous silica nanoparticles with spherical morphology and core-radial mesoporous structure. Characterization included TEM, SEM, HRTEM, XRD, FTIR, TGA, BET surface area, pore size distribution, and GC–MS quantification of loading. Particle size was approximately 220 nm. DMSNs showed high surface area (394.8 m^2^/g) and pore volume (0.861 cm^3^/g); after TEO loading, surface area decreased to 64.4 m^2^/g and pore volume to 0.285 cm^3^/g, confirming incorporation of TEO. Essential oil loading was approximately 15–20%.	Prolonged antifungal activity and the mesoporous structure of DMSNs suggest controlled/sustained release properties. A formal release kinetic study was not reported.	Yes. Free Thyme EO was tested alongside TEO@DMSNs and empty DMSNs controls.	Antifungal activity (antimycotic activity), including fungal growth inhibition, MIC, MFC determination, cell membrane disruption (OD260 leakage), intracellular ROS generation, and morphological damage assessed by SEM.	*Fusarium oxysporum* (plant pathogenic fungus causing Fusarium wilt).	Agricultural/plant protection application. Proposed as a nano-enabled biofungicide/nanofungicide for crop protection. Tomato seedlings were used as a model for plant safety.	− Antifungal assays: TEO@DMSNs incorporated into potato dextrose agar and potato dextrose broth. − Concentrations tested: 0–22 mg/mL for MIC determination. − MIC: 2.75 mg/mL. − MFC: 5.50 mg/mL. − Incubation: *F. oxysporum* was cultured at 28 °C for up to 72 h. − Plant safety: tomato seedlings sprayed with DMSNs (4.95–1.238 mg/mL) and TEO@DMSNs (5.5–1.375 mg/mL) every 3 days for three applications.	Not a food preservation study. Agricultural preservation outcome: inhibition of *F. oxysporum* growth. TEO@DMSNs achieved approximately 69–70% fungal growth inhibition, exceeding free Thyme EO (~38–54% at different time points).	Phytotoxicity/biosafety evaluation using tomato seedlings. No visible wilting or yellowing was observed, and plant height showed no significant differences compared with untreated controls, indicating good crop compatibility at tested concentrations.	Yes. TEO@DMSNs showed superior and sustained antifungal activity compared with free TEO. Example: at 2.75 mg/mL, TEO@DMSNs achieved ~68.79% inhibition after 6 days compared with ~37.70% for free Thyme EO. Encapsulation enhanced ROS generation, membrane disruption, and antifungal persistence.	− Optimization of nanoparticle synthesis conditions required to obtain uniform morphology and reproducible loading. − Need for further development toward practical agricultural application. − Controlled release was inferred but not extensively quantified. − Translation from laboratory antifungal assays to field-scale applications remains a challenge.	Thyme EO-loaded dendritic mesoporous silica nanoparticles provide an effective nano-delivery system that enhances thymol essential oil antifungal activity, improves persistence compared with free Thyme EO, and shows potential as a safe nano-biofungicide for plant protection against *Fusarium oxysporum*.
8 2025 Spain Experimental laboratory-based study [26]	*Allium sativum* L.	Garlic EO-based nano-emulsions prepared using different energy-based methods: (1) RAW emulsion (self-emulsification), (2) SN (sonicated nano-emulsion), (3) SH (sonicated + high-pressure microfluidized nano-emulsion), and (4) HPM (high-pressure microfluidized nano-emulsion). Tween 80 was used as a surfactant.	Characterized by dynamic light scattering. High-energy formulations (SN, SH, HPM) produced nanoscale droplets with low PDI and negative zeta potential. SH and HPM showed the smallest droplet sizes (~72–74 nm after 1 day). HPM maintained nanoscale droplets (~108 nm) after 100 days. PDI values remained low (0.06–0.187) for nano-emulsions, indicating good homogeneity. Stability was monitored over 100 days.	No direct release kinetics or controlled-release study was performed. Stability over time was evaluated by droplet size, PDI, and zeta potential measurements, suggesting physical stability of the nanoemulsions rather than controlled release.	A direct comparison with free garlic EO was not performed. However, different formulation methods were compared. The raw emulsion (larger droplets, poor stability) showed lower insecticidal activity than nano-emulsions.	Insecticidal activity against citrus mealybug (*Planococcus citri*) was evaluated using residual contact toxicity. Mortality, dose–response relationships, LD50, and LD90 values were determined after 24 and 48 h exposure. High-energy nano-emulsions (SN, SH, HPM) achieved 100% mortality at 2.5% EO concentration.	Not applicable. The target organism was an insect pest, not a microorganism. Target pest: *Planococcus citri* (citrus mealybug).	Agricultural plant protection application. The nano-emulsions were developed as botanical biopesticides for controlling citrus mealybug infestations.	Leaf-dip bioassay using citrus leaf discs treated with nano-emulsion formulations. Fifteen third-instar *P. citri* nymphs were exposed per replicate. Mortality was recorded at 24 and 48 h. Each dose was replicated six times. Tested EO concentrations included 0.46%, 0.625%, 0.93%, 1.25%, and 1.87% after preliminary screening.	Not applicable. The study did not evaluate food preservation, shelf life, or postharvest quality. The outcome was pest control efficacy.	Acute toxicity toward beneficial honeybees (*Apis mellifera ligustica*) was evaluated. LD50 and LD90 doses derived from *P. citri* assays were applied topically to bees. After 96 h, nano-emulsion treatments showed no significant mortality compared with the water control, whereas dimethoate caused complete mortality.	No direct comparison with free EO. Compared with the non-nano RAW emulsion, high-energy nano-emulsions provided effective insect control, while the RAW emulsion showed limited activity due to larger droplets and poor stability.	− Maintaining nano-emulsion stability over time. − Selection of energy input method affected droplet size, PDI, and stability. − Raw self-emulsified formulations produced larger, heterogeneous droplets and reduced efficacy. − Heat generation during microfluidization required cooling to prevent EO degradation.	High-energy processing methods successfully generated stable garlic EO nano-emulsions with nanoscale droplets, improved physical stability, and strong insecticidal activity against *Planococcus citri*. The formulations showed selectivity toward the pest while exhibiting low acute toxicity to honeybees, supporting their potential as environmentally friendly botanical biopesticides.
9 2025 China Experimental study [27]	Rose	SL-LNP-stabilized Pickering emulsion (Pickering nanoemulsion) containing Rose EO as the dispersed oil phase.	SL-LNPs were prepared using the solvent diffusion method. SL-LNPs had a mean particle size of 19.62 nm, a PDI of 0.273, and a zeta potential of −32.8 mV. TEM showed spherical nanoparticles. The contact angle was 86.334°, indicating suitable wettability for oil-in-water Pickering emulsions. Optimized Pickering emulsion: 5 mg/mL SL-LNPs, water: oil ratio 10:2, droplet size approximately 259 nm, with good monodispersity and storage stability.	No study reported direct controlled-release or release kinetics. However, the formulation improved Rose EO retention under UV exposure and increased REO bioavailability after simulated digestion.	Compared with a conventional Rose EO emulsion (Tween 80, 2% *v*/*v*). Free Rose EO was not directly tested; instead, a non-nano Rose EO emulsion served as the comparison.	Antioxidant activity: ABTS and DPPH radical scavenging assays. Antimicrobial activity: antibacterial and antibiofilm activity. Other biological outcomes: enhanced Rose EO bioavailability after digestion.	Gram-positive bacteria: *Staphylococcus aureus*, MRSA. Gram-negative bacteria: *Escherichia coli*.	Food preservation and food safety applications. The study proposes the formulation as a natural antimicrobial system to improve food preservation and replace synthetic preservatives.	Antibacterial assay: bacteria (~10^8^ CFU/mL for *S. aureus*, similar treatment for MRSA and *E. coli*) incubated with Rose EO emulsion or SL-LNP Pickering emulsion for 1 h at 37 °C. Biofilm assays: inhibition and disruption assays using 96-well plates, 37 °C incubation. UV stability: emulsions exposed to UV light for 12 h/day; Rose EO retention measured over 7 days. Digestion model: simulated oral (10 min), gastric (2 h), and intestinal (2 h) digestion.	The Pickering emulsion improved the stability and antimicrobial performance of Rose EO. It reduced bacterial survival compared with the Rose EO emulsion: *S. aureus*: survival decreased to 29.75% with Pickering emulsion vs. 55.93% with Rose EO emulsion. *E. coli:* survival decreased to 1.70% with Pickering emulsion vs. 36.83% with Rose EO emulsion. MRSA: survival decreased to 41.05% with Pickering emulsion vs. 61.17% with Rose EO emulsion. The Pickering system inhibited/disrupted biofilm formation and established biofilms more effectively.	Hemolysis assay using mouse erythrocytes. Pickering emulsions showed <5% hemolysis at tested concentrations (15.8–118.5 μg/mL), indicating low hemolytic toxicity and good biocompatibility.	Yes (compared with Rose EO emulsion, not pure free Rose EO). The Pickering emulsion showed: Higher antioxidant activity (ABTS: 82.31% vs. 51.41%; DPPH: 52.76% vs. 28.45%). Stronger antibacterial activity against *S. aureus*, MRSA, and *E. coli*. Greater UV protection (Rose EO retention after 7 days: 41.57% vs. 13.00%). Higher bioavailability (72.18% vs. 47.75%).	The study does not explicitly discuss challenges, but implied challenges include: Need for sufficient SL-LNP concentration to prevent phase separation. Stability dependence on oil/water ratio and particle concentration. Limited stability under extreme pH or high temperatures. Need for further investigation of the interaction mechanism between SL-LNPs and REO.	SL-LNP-stabilized Pickering emulsions effectively encapsulate and protect rose essential oil, improving its antioxidant, antibacterial, antibiofilm activity, UV stability, and gastrointestinal bioavailability while maintaining good biocompatibility. The system shows potential as a natural nano-delivery platform for food preservation applications.
10 2025 China Experimental study [28]	Clove	CEO/AOPs-CSNPs prepared by ionic gelation using chitosan and tripolyphosphate. Tween 80 was used as an emulsifier. Optimal formulation selected: CS:AOPs:Clove EO ratio 1:0.5:0.5.	Particle size: 128.1 ± 1.2 nm (optimal formulation). Zeta potential: 41.7 ± 1.3 mV, indicating good colloidal stability. PDI: 0.119 ± 0.028, indicating a narrow particle distribution. Encapsulation efficiency: 51.31 ± 3.21% for Clove EO and 56.65 ± 3.38% for AOPs. Loading capacity: 16.31 ± 3.2% for Clove EO and 19.56 ± 2.2% for AOPs. Characterization methods: DLS, FTIR, XRD, SEM, TEM.	Controlled release behavior was evaluated by the dialysis method in PBS (pH 7.4, 25 °C). Clove EO release reached 41.40% within 12 h, while AOP release reached 32.16% within 12 h. Release occurred in two phases: initial diffusion-controlled rapid release followed by slower sustained release due to chitosan crosslinking.	CEO/AOPs-CSNPs were compared with free Clove EO, free AOPs, and unloaded CSNPs. The nanoformulation showed higher antioxidant activity, stronger antibacterial effects, improved stability under pH/temperature/UV stress, and better strawberry preservation performance than free Clove EO.	Antioxidant: DPPH radical scavenging, hydroxyl radical scavenging, and FRAP reducing capacity. Antimicrobial: inhibition zones, MIC determination, bacterial morphology analysis, and biofilm inhibition. Stability: antioxidant activity evaluated under different pH, temperature, and UV conditions.	Foodborne bacteria: *Escherichia coli* ATCC 8739, *Staphylococcus aureus* ATCC 25923, and *Pseudomonas aeruginosa* ATCC 9027. Biofilm assays were performed mainly with *E. coli* and *S. aureus*.	Postharvest preservation of strawberries. Strawberries were treated with CEO/AOPs-CSNPs and stored under ambient conditions.	Fresh strawberries were divided into groups: control, Clove EO, AOPs, CSNPs, and CEO/AOPs-CSNPs. Treatments were applied by soaking strawberries for 5 min. Formulation concentration: 5 mg/mL. Storage conditions: 25 °C and 40% relative humidity. Sampling occurred every 2 days until day 8.	CEO/AOPs-CSNPs significantly delayed deterioration. After 8 days, the decay rate was 36.49 ± 2.22% compared with the control (93.29 ± 1.89%). Weight loss was reduced to 6.71 ± 0.35% compared with the control (13.41 ± 0.39%). The formulation preserved ascorbic acid content and maintained stable PPO activity.	Cytotoxicity was evaluated using the MTT assay and live/dead fluorescence staining on L-929 mouse fibroblast cells. Cell viability remained >80% at 100 μg/mL, indicating low cytotoxicity.	Yes. CEO/AOPs-CSNPs showed: higher antioxidant activity than CEO alone; stronger antibacterial effects (larger inhibition zones and lower MIC values); greater biofilm inhibition; improved thermal, pH, and UV stability; and better preservation of strawberry quality compared with free Clove EO.	The study does not explicitly discuss limitations, but potential challenges include moderate encapsulation efficiency (~51% for Clove EO), optimizing EO loading, scaling up nanoparticle production, maintaining stability during storage, and potential regulatory considerations for food applications.	Co-encapsulation of clove essential oil and antioxidant peptide in chitosan nanoparticles produced a stable, controlled-release nano-delivery system with enhanced antioxidant and antimicrobial activity. CEO/AOPs-CSNPs improved strawberry shelf life, preserved nutritional quality, and showed good biocompatibility, indicating potential as a food preservation technology.
11 2025 China Experimental study [29]	Cinnamon	CNEO NEs. Prepared by ultrasonic emulsification using tea saponin as a natural emulsifier.	Optimized formulation: CNEO 3% (*v*/*v*), tea saponin 1.75 mg/mL, sonication power 260 W. Particle size: 169.6 ± 1.1 nm. PDI: 0.197 ± 0.029. Zeta potential: −36.2 ± 0.9 mV. Morphology: spherical droplets with uniform distribution observed by TEM. Nanoemulsion type: oil-in-water (O/W). pH: 5.15 ± 0.05. Viscosity: 30.27 ± 0.25 mPa·s.	Not investigated. No release kinetics or controlled-release profile was reported.	Yes. CNEO nanoemulsion was compared with free CNEO in antimicrobial assays and strawberry preservation experiments. Free CNEO showed lower antimicrobial activity and poorer preservation performance.	Antibacterial activity against *E. coli*; antifungal activity against *Botrytis cinerea* and *Aspergillus flavus*. Assessed by inhibition zones, MIC, MBC/MFC, and mycelial growth inhibition.	Bacteria: *Escherichia coli*. *Fungi*: *Botrytis cinerea* and *Aspergillus flavus*.	Postharvest preservation of strawberries. CEO NEs were applied as edible coatings or dipping treatments to extend shelf life and reduce microbial spoilage.	Fresh strawberries were divided into groups (*n* = 10/group). Treatments included ultrapure water (control), tea saponin (TS), free Cinnamon EO, Cinnamon EO nanoemulsion, and chitosan. Strawberries were immersed for 2 min, dried for 4 h, and stored at ambient temperature (20–25 °C) for 7 days.	CNEO NEs maintained strawberry appearance, reduced weight loss, and delayed fungal spoilage compared with controls. After 7 days, CNEO NE-treated strawberries showed the lowest mass loss (68.17%) and the lowest mildew rate (<40%). CNEO NEs preserved fruit quality better than water, tea saponin, and free Cinnamon EO treatments.	Cytotoxicity was evaluated using an MTT assay in L929 mouse fibroblast cells. CNEO NEs maintained approximately 90% cell viability at concentrations up to 500 μg/mL, suggesting low cytotoxicity under tested conditions.	Yes. Nanoemulsion significantly enhanced antimicrobial activity compared with free CNEO. Against *E. coli*: MIC decreased from 333.75 μg/mL (Cinnamon EO) to 17.81 μg/mL (CNEO NEs). MBC decreased from 667.5 μg/mL to 35.62 μg/mL. Against fungi, CNEO NEs showed substantially lower MIC/MFC values than free Cinnamon EO. Strawberry preservation also improved compared with free cinnamon EO.	Free cinnamon EO has poor water solubility, volatility, and limited stability, which restrict its food applications. The study identified the need to improve the dispersion and stability of EOs through nanoemulsification. Remaining challenges include potential sensory effects of EOs, long-term stability, and further evaluation in different food systems.	CNEO NE prepared with tea saponin provided improved physicochemical stability, enhanced antimicrobial activity, low cytotoxicity, and effective postharvest preservation of strawberries. CNEO NEs represent a promising natural preservative system for food applications.
12 2024 Brazil Experimental in vitro study [30]	*Baccharis dracunculifolia*	Pluronic^®^ F-127 polymeric NP-EO prepared by nanoprecipitation. Control nanoparticles (NP-B) containing capric/caprylic triglyceride and NP-NE were also produced.	Nanoparticles were characterized by dynamic light scattering (particle size, polydispersity index, zeta potential), encapsulation efficiency, pH measurement, and scanning electron microscopy. Particles showed spherical morphology and a mostly monomodal size distribution, with 90% below 200 nm.	No direct release kinetics study was performed. Stability evaluation under different storage temperatures (4, 25, and 37 °C) was used to assess formulation behaviour over time.	Yes. Free EO was tested against EO-loaded nanoparticles using the same antifungal models (agar contact method and volatile exposure).	Antifungal activity was evaluated through mycelial growth inhibition, volatile-phase antifungal activity, nanoparticle antifungal assays, and inhibition of fungal spore germination.	*Botrytis cinerea* and *Rhizopus* sp. were isolated from grape and tomato, respectively.	Potential postharvest preservation and agricultural protection application, targeting fungal pathogens responsible for spoilage of fruits and vegetables. However, validation on real food matrices was not performed.	Free EO, nerolidol, and nanoparticles were tested at concentrations of 1–25 mg/mL in agar contact assays. Volatile activity was tested using 5–25 µL EO/NE applied to filter discs. Nanoparticle antifungal assays evaluated NP-EO and NP-NE at 1–25 mg/mL. Spore germination assays used nanoparticle concentrations of 10, 20, and 25 mg/mL. Incubation: 25 ± 2 °C for up to 7 days.	No direct food preservation experiment. In vitro antifungal protection was demonstrated, with NP-EO inhibiting fungal growth and reducing spore germination of *B. cinerea* and *Rhizopus* sp.	Hemolysis assay using sheep blood. NP-B, NP-EO, and NP-NE showed low hemolytic activity (≈2–3%), suggesting good preliminary biocompatibility.	Partially. Nanoparticles maintained antifungal activity and improved formulation stability, but the study does not clearly demonstrate that NP-EO has superior antifungal efficacy compared with free EO across all assays. Free EO showed strong inhibition against B. cinerea (up to 97.5% at 25 mg/mL), while NP-EO showed up to 87.6% inhibition. The main improvement was attributed to improved formulation stability and controlled-release potential.	No explicit challenges were discussed. Observed limitations include temperature-dependent changes in nanoparticle size, the lack of real-food matrix testing, and no evaluation of EO release kinetics.	Pluronic^®^ F-127 nanoparticles successfully encapsulated *B. dracunculifolia* EO, producing stable nanosystems with antifungal activity against important postharvest fungi and low hemolytic toxicity. The nanoformulation represents a promising strategy for improving EO applicability in agricultural and postharvest protection, although further validation in food systems is required.
13 2024 China Experimental study evaluating [31]	*Litsea cubeba*	*Litsea cubeba* EO and LNE prepared using Tween 80 as surfactant, ethanol as co-solvent, and water as continuous phase. The nanoemulsion was subsequently incorporated into KGM edible coating solutions.	Nanoemulsion droplet size: 66.86 ± 0.14 nm initially. Droplet size, zeta potential, and PDI evaluated during storage (0, 3, 6, and 12 days). Zeta potential: negative values (approximately −3.9 to −18.7 mV depending on formulation). PDI measured to assess droplet size distribution and stability. Morphology and distribution evaluated by CLSM and SEM. Maximum droplet size after 12 days: 139.53 ± 1.27 nm, maintaining homogeneity	*Litsea cubeba* EO retention and release from dried coatings were evaluated. *Litsea cubeba* EO retention increased with increasing LNE content (20.87–47.47% in fresh coatings). Retention decreased during storage (18 days). *Litsea cubeba* EO showed an initial rapid release followed by sustained release. Release was evaluated in deionized water and 95% ethanol-water food simulants. Faster release occurred in aqueous conditions due to KGM swelling/dissolution.	Free *Litsea cubeba* EO (dissolved in 50% ethanol-water solution) was compared with LNE. The nanoemulsion showed stronger antimicrobial activity than free *Litsea cubeba* EO.	Antibacterial activity. Antifungal activity. Antimicrobial preservation effect during citrus storage. Nanoemulsion enhanced antimicrobial performance compared with free essential oil.	*Escherichia coli* O157:H7 *Staphylococcus aureus* *Penicillium* italicum (citrus postharvest pathogen)	Postharvest preservation of citrus fruit using LNE-containing konjac glucomannan edible coatings. Application aimed at reducing microbial spoilage and maintaining fruit quality	LNE incorporated into KGM coatings at different concentrations: 1.25, 2.5, 5, and 10 g LNE/100 g coating solution. Citrus fruits immersed in coating solutions for 120 s. Fruits stored at 15 ± 2 °C and 70 ± 2% RH.	Reduced microbial growth (lower total viable counts). Delayed citrus decay caused by *P. italicum*. Reduced weight loss. Maintained fruit hardness. Preserved vitamin C content. Improved retention of soluble solids and acidity.	Phytotoxicity was evaluated during citrus application. High LNE concentration (KGM-LNE 10) caused oleocellosis and tissue damage, indicating phytotoxic effects. KGM-LNE 5 provided antifungal activity without evident phytotoxicity.	Yes. LNE exhibited significantly larger inhibition zones against *E. coli*, *S. aureus*, and *P. italicum* compared with free *Litsea cubeba* EO. Nanoemulsification enhanced antimicrobial activity, likely because it improved dispersion and the availability of EO compounds.	High EO concentrations may induce phytotoxicity. Nanoemulsion stability decreases over time due to droplet growth/Ostwald ripening. Excess LNE may weaken coating structure and increase water/oxygen permeability. Volatility and degradation of EO components may reduce antimicrobial activity during storage. Optimization of EO concentration is necessary to balance efficacy and safety.	LNE incorporated into konjac glucomannan edible coatings effectively enhanced antimicrobial activity and improved citrus preservation. The nanoemulsion provided controlled release and improved LCO stability, but excessive concentrations caused phytotoxicity. The optimized formulation (particularly KGM-LNE 2.5) represents a promising nano-enabled edible coating strategy for postharvest food preservation.
14 2024 Italy Experimental laboratory study [32]	*Allium sativum* L.	Garlic EO nano-emulsion prepared by high-pressure microfluidisation. Composition: EO 15% (*w*/*w*), Tween 80 surfactant 5% (*w*/*w*), water 80% (*w*/*w*).	Characterized by dynamic light scattering. Particle size: 64.5 ± 0.51 nm (24 h), 82.8 ± 0.45 nm (3 months), 221.4 ± 2.67 nm (1 year). PDI: 0.05 ± 0.01 to 0.17 ± 0.02, indicating relatively homogeneous particle distribution. Zeta potential: −17.6 ± 1.25 mV (24 h), decreasing to −30.4 ± 0.52 mV after one year. Chemical composition assessed by SPME-GC-MS.	Not evaluated. No controlled-release, release-kinetics, diffusion, or release-profile experiments were reported.	Chemical composition of crude garlic EO and nano-emulsified EO was compared by GC-MS. However, the authors did not directly compare biological efficacy with free EO in insect bioassays.	Insecticidal activity against citrus mealybug (*Planococcus citri*). Evaluated by direct contact toxicity and residual contact toxicity. LC50 and LC90 values were determined after 24 and 48 h.	Not applicable. No microorganisms were evaluated.	Agricultural application: nano-emulsion developed as a biopesticide for control of *Planococcus citri* in citrus production systems.	Laboratory bioassays at 28 ± 1 °C, 70 ± 5% relative humidity, 12:12 h light: dark photoperiod. Nano-emulsion applied by spraying (direct exposure) and leaf-dip treatment (residual exposure). Tested concentrations varied by assay. Mortality assessed at 24 and 48 h.	Not applicable. No food preservation or postharvest preservation outcomes were assessed.	Acute toxicity was evaluated against the beneficial predator *Cryptolaemus montrouzieri*. Survival after exposure to the LC90 and maximum dose of garlic EO nano-emulsion was assessed over three days. No significant mortality was observed compared with the untreated control, indicating low toxicity toward the non-target organism.	Not demonstrated. The study did not include a free EO treatment group in the insecticidal assays; therefore, the enhanced efficacy of the nanoformulation cannot be directly confirmed.	Nano-emulsion stability over time was a concern. Particle size increased from 64.5 nm after 24 h to 221.4 nm after one year, indicating gradual physicochemical changes during storage. No major formulation instability was observed based on PDI and zeta potential measurements.	Garlic EO nano-emulsion showed strong insecticidal activity against *Planococcus citri* while maintaining low toxicity toward the beneficial predator *Cryptolaemus montrouzieri*. The nanoformulation represents a promising, sustainable biopesticide approach, although a direct comparison with free EO is needed to confirm the nano-enabled efficacy enhancement.
15 2023 India Experimental study [33]	*Melaleuca cajuputi*	CjEO-CSNP. Prepared by ionic gelation using chitosan as the polymer matrix and sodium tripolyphosphate as a cross-linking agent.	− Carrier: low molecular weight chitosan (>75% deacetylation degree). − Preparation method: ionic gelation with oil-in-water emulsion formation. − Optimal formulation: chitosan:CjEO ratio 1:1 (*w*/*v*). − Encapsulation efficiency: 92.26%. − Loading efficiency: 8.87%. − Particle size: ~77.13 ± 1.48 nm for optimized CjEO-CSNP. − ζ-potential: +27.06 to +44.33 mV depending on EO concentration. − PDI: 0.11–0.25, indicating a relatively narrow particle size distribution. − Morphology: spherical nanoparticles confirmed by SEM. − Structural changes confirmed by XRD after EO incorporation.	Controlled release was evaluated in acetate buffer (pH 3) and phosphate-buffered saline (pH 7). Biphasic release profile observed: rapid initial release during the first 24 h (82.92% in acetate buffer; 73.42% in phosphate buffer), followed by slower sustained release up to 168 h. Release was greater under acidic conditions due to chitosan swelling/dissolution.	Direct comparison with free *Melaleuca cajuputi* EO was not performed in the mushroom preservation experiment. The study referenced previous toxicity data for free EO (LD50 = 1600 mg/kg), but it did not conduct comparative efficacy assays between free EO and CjEO-CSNP.	Antioxidant activity was evaluated by DPPH and ABTS radical scavenging assays. CjEO-CSNP coating enhanced antioxidant preservation of mushrooms and maintained total phenolic content during storage. Also improved preservation-related parameters (reduced respiration rate, reduced weight loss, improved sensory quality).	Not directly evaluated. No antimicrobial assays (MIC, MBC, inhibition zones, microbial counts) were performed. Microbial inhibition was only suggested as a possible mechanism.	Postharvest food preservation. CjEO-CSNP was applied as a coating to fresh button mushrooms (*Agaricus bisporus*) to extend shelf life.	Mushrooms were divided into two groups: 1. Control: chitosan nanoparticle coating (CSNP). 2. Treatment: CjEO-loaded chitosan nanoparticle coating (CjEO-CSNP). Mushrooms were stored at 4 °C for 7 days. Quality was evaluated on day 3 and day 7.	CjEO-CSNP coating resulted in: − Reduced weight loss compared with control. − Lower respiration rate. − Better maintenance of mushroom appearance and firmness. − Higher antioxidant activity (DPPH/ABTS). − Greater retention of total phenolic compounds. − Improved sensory attributes (odor, color, surface uniformity, overall acceptability). − Extended freshness during refrigerated storage.	Acute oral toxicity study in Swiss albino male mice. Tested doses of 200–2000 mg/kg body weight. No toxicity observed up to 1200 mg/kg. Toxic effects and mortality occurred at higher doses. LD50 estimated by Probit analysis.	Limited. The study demonstrated improved performance compared with chitosan nanoparticles without EO (CSNP control), but did not directly compare CjEO-CSNP with free *Melaleuca cajuputi* EO for preservation efficacy. Therefore, improvement over free EO cannot be confirmed.	− Essential oil volatility and susceptibility to oxidation require encapsulation. − EO loading affects particle size and ζ-potential. − Stability depends on polymer/cross-linker concentrations and formulation conditions. − Acidic pH influences chitosan swelling and release behavior. − Sensory impact of EO release needs control to avoid undesirable odor.	CjEO-CSNP produced by ionic gelation provided efficient nanoencapsulation, controlled EO release, antioxidant activity, and improved preservation of button mushrooms during refrigerated storage. The formulation showed potential as an active food coating system with acceptable acute oral safety, although a direct comparison with free-essential-oil efficacy remains lacking.
16 2023 Italy Experimental study [34]	*Thymus vulgaris* L.	CNCs@TEO nanoemulsion: oil-in-water Pickering nanoemulsion stabilized by CNCs, with Tween 80 as surfactant, ethanol as cosurfactant, and CaCl_2_ as ionic crosslinker. The optimized formulation was CNCs@TEO NE_1, containing 10% *v*/*v* thyme EO, CNCs, and CaCl_2_.	− Nanoemulsion type: CNC-stabilized Pickering nanoemulsion − Oil retention/encapsulation efficiency: 100% − Droplet size (DLS Z-average): ~230 nm − TEM droplet diameter: 109.4 ± 18 nm − PDI: <0.4, indicating homogeneous particle distribution − ζ-potential: −25 to −27 mV − Morphology: spherical and homogeneous droplets − Stability assessed by DLS, centrifugation, SI index, and Turbiscan analysis	No formal release kinetics study was performed. The study demonstrated controlled retention/stabilization of thyme EO within the nanoemulsion. The authors observed delayed EO availability after application, suggesting reduced volatilization and gradual antimicrobial action compared with free EO.	Yes. Free thyme EO was compared with CNCs@TEO NE_1 using antimicrobial assays. The nanoemulsion showed enhanced antimicrobial performance, including larger inhibition halos and improved biological persistence.	Antibacterial activity was evaluated through: − Agar incorporation assay − Disc diffusion assay − Microdilution assay − In planta epiphytic survival assay The nanoemulsion inhibited bacterial growth and reduced pathogen survival on olive leaves.	*Pseudomonas savastanoi* pv. *savastanoi* (Psav), the causal agent of olive knot disease.	Agricultural plant protection/biopesticide application. The nanoemulsion was evaluated as an alternative antimicrobial treatment to protect olive plants against bacterial disease.	− EO concentrations tested: 0.05–1% *v*/*v* in antimicrobial assays − Optimized NE tested at 0.5% *v*/*v* on olive plants − Plants treated by foliar spray until runoff − Pathogen inoculation after 24 h − Bacterial survival monitored at 1, 7, and 14 days post-inoculation − Copper sulfate (0.3% *w*/*v*) used as positive control; water as negative control	Not a food preservation study. Agricultural preservation outcome: − Reduced survival of *P. savastanoi* on olive leaves − After 7 and 14 days, CNCs@TEO NE achieved bacterial reduction comparable to copper sulfate treatment − Maintained antimicrobial activity over time, suggesting potential for plant disease management.	Phytotoxicity evaluation in olive seedlings: − Chlorophyll content − Flavonol content − Nitrogen balance index − Leaf development/area measurements No adverse effects were observed following treatment with components of the thyme EO nanoemulsion.	Yes. Evidence includes: − Nanoemulsion produced larger inhibition halos than free EO in disc diffusion assays. − Encapsulation reduced EO volatilization/dispersion and improved delivery. − Nanoemulsion maintained antimicrobial activity over longer periods on olive plants compared with the expected rapid loss of free EO.	− EOs are volatile and chemically unstable, requiring stabilization. − Increasing EO concentration can compromise nanoemulsion stability. − Need to optimize EO/CNC ratio and crosslinking conditions. − Long-term field stability remains uncertain due to environmental factors (light, temperature, humidity, precipitation). − Further field-scale validation is required.	CNC-stabilized Pickering nanoemulsions provide an effective delivery strategy for thyme essential oil, improving physical stability, reducing EO volatilization, maintaining antimicrobial activity, and enabling agricultural application as a potential biopesticide against *Pseudomonas savastanoi* pv. *savastanoi* while avoiding phytotoxic effects on olive plants.
17 2022 China Experimental study [35]	Oregano	Oregano essential oil nanoemulsion prepared using Tween 80 as a non-ionic surfactant and ultrasound-assisted emulsification. Optimized formulation: 4% (*w*/*v*) Oregano EO nanoemulsion with 20 min sonication.	Characterized by dynamic light scattering. Optimized Oregano EO nanoemulsion showed particle size ≈ 153 nm, PDI ≈ 0.42, and ζ-potential ≈ −23.5 mV. Nanoemulsions showed acceptable centrifugation stability; 6% Oregano EO formulations showed phase separation and were excluded.	Oregano EO retention/loss from chitosan films was evaluated during storage at room temperature for 6 days. Higher Oregano EO concentrations (CS-1%OEOF and CS-1.5%OEOF) showed greater Oregano EO loss, suggesting faster release from films. No detailed release kinetics or diffusion modelling were performed.	Yes. Free Oregano EO was compared with Oregano EO nanoemulsion during antifungal screening. Nanoemulsions showed enhanced antifungal activity compared with lower-concentration/non-sonicated Oregano EO formulations.	Antifungal activity against Alternaria alternata; inhibition of fungal growth; reduction in apricot black spot disease incidence and lesion development.	*Alternaria alternata* (isolated from infected apricot fruit).	Active food packaging/postharvest preservation of apricot (*Prunus armeniaca* L.). Oregano EO nanoemulsion was incorporated into chitosan films applied to the inside of fruit storage containers.	Apricots were inoculated with A. alternata spores (1 × 10^5^ spores mL^−1^, 20 µL per wound). Fruits were stored at 20 ± 1 °C and 90–95% RH. Films containing 0, 0.2, 0.5, 1, and 1.5% (*v*/*v*) Oregano EO nanoemulsion were tested. Disease parameters were evaluated after 3 and 6 days.	CS-OEO films significantly reduced black spot development. CS-1%OEOF and CS-1.5%OEOF showed the strongest antifungal effect, reducing lesion diameter and disease incidence compared with untreated control and chitosan-only films. No significant difference was observed between 1% and 1.5% Oregano EO formulations, indicating that 1% is the optimal concentration.	Zebrafish (*Danio rerio*) toxicity assay. Fish exposed to CS-1%OEOF films maintained high viability after 12 days, suggesting low acute toxicity of the active film formulation.	Yes, partially. Nanoemulsification improved antifungal activity compared with free Oregano EO in screening assays, attributed to smaller droplet size, increased surface area, and improved dispersion. However, direct quantitative comparison of equivalent EO concentrations between free EO and nanoencapsulated EO in fruit preservation was limited.	High Oregano EO concentrations negatively affected nanoemulsion stability (higher PDI and phase separation). Incorporation of nanoemulsions altered film microstructure, increasing porosity. Higher Oregano EO loading accelerated EO loss during storage. Need for improved release control and long-term stability evaluation.	Ultrasound-assisted Oregano EO nanoemulsions incorporated into chitosan films provided effective antifungal activity and controlled apricot black spot disease during postharvest storage. The CS-OEO nanoactive films showed potential as sustainable active packaging materials with acceptable preliminary safety.
18 2020 India Experimental laboratory study [36]	*Monarda citriodora*	*Monarda citriodora* EO-loaded chitosan nanoparticles prepared using an oil-in-water emulsion followed by tripolyphosphate ionic cross-linking. Chitosan served as the polymeric nanocarrier, and Tween-80 as the emulsifier.	− Nanoparticle type: chitosan nanoparticles (polymeric nanoparticles). − Particle size: 25.14–58.45 nm for *Monarda citriodora* EO-loaded nanoparticles (SEM). − Shape: nearly spherical; some agglomeration observed. − Encapsulation efficiency (EE): 61.25 ± 4.07%. − Loading capacity (LC): 2.10 ± 0.14%. − Characterization techniques: SEM, FTIR, XRD. − FTIR and XRD confirmed successful incorporation of *Monarda citriodora* EO into the chitosan matrix.	No formal release kinetics or controlled-release profile was evaluated. The authors suggested improved stability and sustained release as possible mechanisms for enhanced activity, but they did not provide quantitative release data.	Direct comparison was performed between free *Monarda citriodora* EO and *Monarda citriodora* EO-loaded chitosan nanoparticles. Free *Monarda citriodora* EO required higher concentrations for inhibition compared with the nanoformulation.	Activities evaluated: − Antifungal activity. − Antiaflatoxigenic activity (inhibition of aflatoxin B1 production). − Antioxidant activity (DPPH assay). − Antifungal mechanism studies: ergosterol inhibition, membrane damage (PI staining/flow cytometry), leakage of cellular ions and intracellular materials, methylglyoxal inhibition.	Main target: *Aspergillus flavus* AF-LHP-SH1 (high aflatoxin-producing strain). Additional fungal species tested: *A. luchuensis*, *A. niger*, *A. sydowii*, *A. repens*, *A. fumigatus*, *A. chevalieri*, *A. terreus*, *Alternaria grisea*, *A. humicola*, *A. versicolor*, *Fusarium oxysporum*, *Penicillium italicum*, *P. spinolosum*, *A. alternata* and mycelia sterilia.	Food preservation application, particularly protection of stored functional foods (seeds) against fungal contamination and aflatoxin B1 production. Tested on functional food commodities including flaxseed, barley, chia, buckwheat, sesame, horse gram and quinoa. Also evaluated potential agricultural relevance through seed germination testing.	Free *Monarda citriodora* EO: − Antifungal assay concentrations: 0.20–1.40 µL/mL. − MIC: 1.40 µL/mL against *A. flavus*. − Complete AFB1 inhibition at 1.20 µL/mL. *Monarda citriodora* EO-loaded chitosan nanoparticles: − Tested concentrations: 0.10–0.60 µL/mL equivalent *Monarda citriodora* EO. − Antifungal activity evaluated in SMKY medium against *A. flavus*. − Incubation: 27 ± 2 °C for 10 days.	− Free *Monarda citriodora* EO completely inhibited fungal growth at 1.40 µL/mL. − Free *Monarda citriodora* EO completely inhibited AFB1 production at 1.20 µL/mL. − Nanoencapsulated *Monarda citriodora* EO inhibited fungal growth at 0.60 µL/mL. − Nanoencapsulated *Monarda citriodora* EO inhibited AFB1 production at 0.50 µL/mL. − Nanoformulation reduced the effective concentration required for antifungal and antiaflatoxigenic activity.	Phytotoxicity was evaluated using Salvia hispanica seeds stored with *Monarda citriodora* EO for 6 months. Germination percentage, radicle length, and plumule length were assessed. *Monarda citriodora* EO treatment showed no adverse effect on seed germination. No cytotoxicity, ecotoxicity, or human safety assessment was reported.	Yes. *Monarda citriodora* EO-loaded chitosan nanoparticles showed approximately 2-fold higher antifungal and antiaflatoxigenic efficacy compared with free *Monarda citriodora* EO: − Antifungal MIC decreased from 1.40 µL/mL (free EO) to 0.60 µL/mL (nanoformulation). − AFB1 inhibition concentration decreased from 1.20 µL/mL (free EO) to 0.50 µL/mL (nanoformulation).	The authors identified limitations of free EO application: − High volatility. − Susceptibility to degradation by light, oxygen, moisture, and temperature. − Flavor limitations in food applications. − Poor stability during large-scale application. A nanocarrier formulation was proposed to improve EO stability, retention, and delivery.	*Monarda citriodora* EO-loaded chitosan nanoparticles represent a promising nano-enabled food preservation strategy. Nanoencapsulation improved antifungal and antiaflatoxigenic activity compared with free EO and may provide an effective approach for controlling fungal contamination and aflatoxin B1 formation in stored functional foods. Further studies on food application, safety evaluation, and large-scale implementation are required.
19 2019 China Experimental research study [37]	Laurel	Liposomal nano-delivery system: Lip-LEO and Lip-LEO-AgNPs. Laurel EO was encapsulated in the hydrophobic bilayer region of liposomes, while hydrophilic lignin-AgNPs were incorporated into the aqueous phase. The final formulation was incorporated into a chitosan coating applied to polyethylene films (PC-Lip/LEO/AgNPs).	Lip-LEO-AgNPs showed spherical morphology. Characterization included TEM, SEM, HAADF imaging, EDS, UV–vis spectroscopy, particle size analysis, PDI, zeta potential, and encapsulation efficiency. Mean particle size: ~200 nm. PDI: 0.27, indicating a relatively uniform distribution. Zeta potential: −26 mV, suggesting colloidal stability. Encapsulation efficiency: LEO 68%; AgNPs 57%.	Liposomes released Laurel EO and AgNPs in a controlled manner. LEO release reached ~29.3% after 168 h in dialysis conditions, indicating delayed release. AgNP release was limited (~11.79% after 168 h in PBS dispersion), indicating effective retention of nanoparticles within liposomes.	Direct comparison with free Laurel EO was not clearly performed. The authors compared liposomal formulations (Lip-Laurel EO and Lip-LEO-AgNPs) mainly with blank liposomes and PE/PE-CS packaging films. The authors reported that encapsulation slowed Laurel EO release compared with dispersed/free conditions.	Antioxidant activity was assessed by DPPH radical scavenging assay, FRAP reducing power assay, and ABTS/TRAP assay. Antimicrobial activity was evaluated using agar plate method. Lip-LEO-AgNPs exhibited antioxidant capacity and strong antibacterial activity.	*Staphylococcus aureus* (Gram-positive bacteria) and *Escherichia coli* (Gram-negative bacteria).	Active food packaging to preserve fresh pork stored under refrigeration (4 °C).	Pork samples (1480 g total) were divided into groups: untreated control, PE film, PE-CS film, and PC-Lip/LEO/AgNPs film. Samples were stored at 4 °C and evaluated over 0, 3, 6, 9, 12, 15, 18, and 21 days. Films were prepared by casting 5 mL chitosan/liposome coating solution onto PE films and drying at 40 °C.	PC-Lip/LEO/AgNPs films extended pork shelf life compared with controls. Sensory quality remained acceptable longer (up to ~15 days). TVB-N values exceeded acceptable limits later in the coated samples than in the control. Packaging reduced pH increase and delayed spoilage.	Cytotoxicity was evaluated using L02 human hepatocyte cells exposed to pork extracts from packaged samples. Cell viability remained >90% during storage; viability remained >80% even at 30% extract concentration. Live/dead fluorescence staining confirmed low cytotoxicity. ICP-OES measured silver migration into pork; final Ag content was low (~3.2 μg/L).	Partial evidence only. Encapsulation improved controlled release and stability of Laurel EO, but the study did not directly compare free EO with nanoencapsulated EO. Enhanced preservation efficacy was demonstrated compared with PE and PE-CS films, but the contribution of Laurel EO alone cannot be separated from that of AgNPs and chitosan.	Need to control AgNP release to minimize potential toxicity; difficulty in separating the contribution of EO, AgNPs, and chitosan to antimicrobial effects; requirement for improved encapsulation efficiency and controlled delivery. Scale-up and application compatibility of nano-enabled packaging remain challenges.	Laurel EO-loaded liposomes combined with lignin-AgNPs, incorporated into chitosan-coated PE films, provided controlled release, antioxidant and antibacterial activities, and improved refrigerated pork preservation. The nano-enabled active packaging system demonstrated potential to extend food shelf life while maintaining low cytotoxicity.

ABTS: 2,2′-azino-bis(3-ethylbenzothiazoline-6-sulfonic acid); AOP: Antioxidant peptides; CEO/AOP-CSNPs: Clove EO/AOPs-loaded chitosan nanoparticles; CjEO-CSNP: *Melaleuca cajuputi* EO-loaded chitosan nanoparticles; CNCs: Cellulose nanocrystals; DMSNs: Dendritic mesoporous silica nanoparticles; DPPH: 2,2-diphenyl-1-picrylhydrazyl; EDS: Energy dispersive spectroscopy; EO: Essential oil; EON: Essential oil nanoemulsion; FRAP: Ferric ion reducing antioxidant power; GC-MS: Gas chromatography–mass spectrometry; HAADF: Highangle Annular Dark Field; KC: κ-carrageenan; KGM: Konjac glucomannan; LIP-TEO-CuNPs: Liposome-based nano-delivery system containing thyme essential oil and copper nanoparticles; LNE: *Litsea cubeba* nanoemulsion; MBC: Minimum bactericida concentration; MFC: Minimum fungicidal concentration; MIC: Minimum inhibitory concentration; MRSA: Methicillin-resistant *S. aureus*; MTT: 3-(4,5-Dimethylthiazol-2-yl)-2,5-Diphenyltetrazolium Bromide; Ne-CEO: Coriandum-loaded chitosan nanoemulsion; NEO: Nanoemulsion; NP-EO: Nanoparticles containing EO; NP-NE: Nerolidol-loaded nanoparticles; PDI: Polydispersity index; SA: Sodium alginate; SEM: Scanning electron microscope; SL-LNP: Soybean lecithin lipid nanoparticle; TBARS: Thiobarbituric acid reactive substances; TEM: transmission electron microscope; TEO@DMSNs: Thyme EO-loaded dendritic mesoporous silica nanoparticles; XRD: X-ray diffraction.

**Table 3:** Synthesis of safety outcomes reported for nanoencapsulated essential oils included in the systematic review.

Safety Outcome	Studies (*n*)	Main Findings	Overall Evidence	Remaining Gaps
Cytotoxicity	7	Most nanoformulations maintained high cell viability (>80–90%) at the tested concentrations in fibroblast or mammalian cell lines.	Generally favourable	Long-term cytotoxicity, repeated exposure, and multiple human cell models remain largely unexplored.
Hemolysis	3	Hemolysis was consistently low (<5%), indicating good blood compatibility of the tested formulations.	Low acute hemolytic toxicity	No studies evaluated in vivo hematological effects.
Phytotoxicity/Plant compatibility	3	Plants tolerated most formulations at optimized concentrations. However, excessive EO loading occasionally caused leaf damage or oleocellosis.	Dose-dependent safety	Species-specific phytotoxicity thresholds require further investigation.
Sensory evaluation	2	Nanoencapsulation reduced undesirable sensory changes while maintaining acceptable appearance, aroma, and overall quality of treated foods.	Promising	Consumer acceptance studies remain limited.
Nanoparticle migration	1	Migration from packaging materials into food simulants was minimal and remained below proposed safety limits.	Favourable preliminary evidence	Additional studies under different storage conditions and food matrices are needed.
Ecotoxicity/Non-target organisms	1	Garlic EO nanoemulsions showed low acute toxicity toward honeybees compared with conventional pesticide controls.	Encouraging	Long-term ecological effects and environmental persistence remain unknown.
Overall safety	19	The included studies reported no severe acute toxicity when formulations were used at the investigated concentrations.	Generally favourable short-term safety profile	Chronic toxicity, biodistribution, bioaccumulation, environmental fate, reproductive toxicity, and regulatory toxicology data are lacking.

EO: Essential oil.

**Table 4:** Synthesis of technological outcomes reported for nanoencapsulated essential oils included in the systematic review.

Technological Aspect	Overall Findings	Main Challenges/Knowledge Gaps	Supporting References
Particle size and colloidal stability	Most nanoformulations produced nanoparticles or droplets < 250 nm, with low polydispersity indices and adequate zeta potentials, indicating good physical stability.	Long-term storage stability under commercial conditions requires further investigation.	[19,20,21,22,23,24,25,26,27,28,29,30,31,32,33,34,35,36]
Encapsulation efficiency	Most formulations achieved moderate to high encapsulation efficiency, improving protection of volatile compounds from degradation.	Encapsulation efficiency varied considerably depending on carrier materials and preparation methods.	[19,20,21,22,23,24,25,26,27,28,29,30,31,32,33,34,35,36]
Controlled/sustained release	Several studies demonstrated biphasic or sustained release profiles that prolonged antimicrobial activity and reduced EO loss.	Standardized release kinetics were lacking, and many studies inferred controlled release without formal evaluation.	[21,22,24,25,27,29,31,32,33,36]
Physicochemical stability	Nanoencapsulation improved resistance to oxidation, UV exposure, temperature, and pH changes while reducing EO volatility.	Stability under industrial processing and prolonged storage remains insufficiently studied.	[19,20,21,22,23,24,25,26,27,28,29,30,31,32,33,34,35,36]
Antimicrobial persistence	Sustained release often resulted in longer antimicrobial and antifungal activity than free EOs.	Validation under real food-processing and field conditions is still needed.	[20,21,22,23,24,25,26,27,28,29,30,31,32,33,34,35,36]
Food preservation performance	Nanoformulations effectively delayed spoilage, reduced microbial growth, maintained physicochemical quality, and extended shelf life of several fresh products.	Most studies evaluated only a limited number of food matrices and storage conditions.	[19,21,22,23,24,28,29,30,31,32,33,35,36]
Agricultural performance	Nanoencapsulated EOs effectively controlled plant pathogens and insect pests while generally maintaining acceptable crop compatibility.	Large-scale field trials and environmental performance remain scarce.	[25,26,34,36]
Scalability and industrial implementation	Nanoencapsulation technologies show strong laboratory potential for food and agricultural applications.	Industrial-scale production, cost-effectiveness, reproducibility, quality control, and regulatory approval remain major barriers to commercialization.	[19,20,21,22,23,24,25,26,27,28,29,30,31,32,33,34,35,36]

EOs: Essential oils; UV: Ultraviolet.

**Table 5:** Methodological quality assessment of the included studies. Studies were evaluated using an 11-item modified quality assessment checklist, with each item scored as 0 (not reported), 1 (partially reported), or 2 (fully reported). Overall methodological quality was assessed using the QUIN percentage score and classified as low (green), moderate (orange) or high (red).

Study No.	S1	S2	S3	S4	S5	S6	S7	S8	S9	S10	S11	Final Score/QUIN Score (%)	Quality Classification
1	2	0	1	2	0	0	0	2	2	2	2	13/59.1	Moderate
2	2	0	2	2	0	0	0	2	2	2	2	14/63.6	Moderate
3	2	0	1	2	0	0	0	2	1	2	1	11/50.0	Moderate
4	2	0	1	2	2	0	0	2	2	2	2	15/68.2	Moderate
5	2	0	2	2	0	0	0	2	2	2	2	14/63.6	Moderate
6	2	0	2	2	0	0	2	2	2	2	2	16/72.7	Low
7	2	0	1	2	0	0	0	2	1	2	2	12/54.5	Moderate
8	2	0	1	2	0	0	0	2	1	2	2	12/54.5	Moderate
9	2	0	1	2	0	0	0	2	1	2	2	12/54.5	Moderate
10	2	0	1	2	1	0	0	2	2	2	2	14/63.6	Moderate
11	2	0	1	2	1	0	0	2	1	2	2	13/59.1	Moderate
12	2	0	1	2	0	0	0	2	2	2	2	13/59.1	Moderate
13	2	0	1	2	0	0	0	2	2	2	2	13/59.1	Moderate
14	2	0	1	2	0	0	0	2	2	2	2	13/59.1	Moderate
15	2	0	1	2	1	0	1	2	2	2	2	15/68.2	Moderate
16	2	0	2	2	0	0	0	2	2	2	2	14/63.6	Moderate
17	2	0	1	2	2	0	0	2	2	2	2	15/68.2	Moderate
18	2	0	2	2	1	0	0	2	2	2	2	15/68.2	Moderate
19	2	0	1	2	1	0	0	2	1	2	2	13/59.1	Moderate

S1: Clearly states aims and objectives; S2: Detailed explanation of sample size calculation; S3: Detailed explanation of sampling techniques; S4: Detailed description of experimental conditions; S5: Randomization; S6: Allocation concealment; S7: Blinding of investigators/outcome assessors; S8: Outcome measurement reliability; S9: Appropriate statistical analysis; S10: Reporting of results; S11: Conclusions supported by data.

## Data Availability

No new data were created or analyzed in this study. Data sharing is not applicable to this article.

## Abbreviations

The following abbreviations are used in this manuscript:

EOs	Essential oils
PICOS	Population, Intervention, Comparator, Outcomes, and Study design
PRISMA	Preferred Reporting Items for Systematic reviews and Meta-Analyses
QUIN	Quality Assessment Tool for In Vitro Studies
